# Kosmotrope-Promoted Proton Hopping in Supramolecular Conductors

**DOI:** 10.1021/jacs.6c03867

**Published:** 2026-05-14

**Authors:** Wei-Bin Lin, Yongjiu Lei, Pei Yu, Lukman O. Alimi, Jinrong Wang, Basem A. Moosa, Niveen M. Khashab

**Affiliations:** † Smart Hybrid Materials Laboratory (SHMs), Chemistry Program, Physical Science and Engineering Division, 127355 King Abdullah University of Science and Technology (KAUST), Thuwal 23955-6900, Saudi Arabia; ‡ Materials Science and Engineering, Physical Science and Engineering Division, King Abdullah University of Science and Technology (KAUST), Thuwal 23955-6900, Saudi Arabia

## Abstract

Scientific interest in the Hofmeister effect has waxed and waned since its discovery over a century ago; however, its ion-specific influence on solvation and molecular organization remains a topic of enduring significance. While extensively studied in aqueous systems, its relevance to solid-state materials and Grotthuss mechanisms has remained largely unexplored. Here, we unveil a class of supramolecular organic cage salts, the Cage1 series, with counteranions spanning the Hofmeister series to systematically probe their ion-specific effects on solid-state proton conduction. Kosmotropic anions, stabilized by the supramolecular organic cage, are found to markedly enhance proton conductivity, promoting the formation of an extended hydrogen-bonded network (HB-network) that facilitates Grotthuss-type proton hopping. The conductivity increases by over 2 orders of magnitude from chaotropic Cage1-HI to kosmotropic Cage1-H_2_SO_4_, reaching up to 1.03 × 10^–1^ S cm^–1^ at 333 K under 95% RH. Single-crystal and molecular dynamics (MD) analyses reveal how distinct cage–anion–water interactions modulate proton mobility at the molecular level. These findings establish an unprecedented link between Hofmeister chemistry and proton transport in solids, offering a new ion-specific design principle for next-generation supramolecular proton conductors.

## Graphical abstract

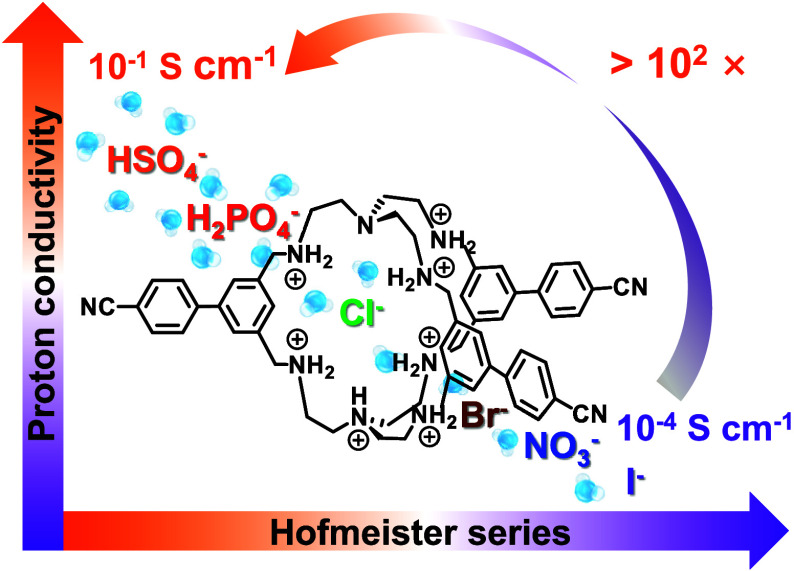

## Introduction

With the growing need to address global energy consumption while advancing clean and sustainable energy technologies, the development of high-performance proton exchange membranes (PEMs) has emerged as a key component in diverse energy storage and conversion systems.^1,2^ Enhancing proton conductivity remains a central goal for boosting the power output of practical, sustainable systems. A broad spectrum of PEM materials, including covalent–organic frameworks (COFs),^3–9^ metal–organic frameworks (MOFs),^10–12^ porous organic polymers,^13,14^ and salts,^15–18^ engage in varying degrees of interaction with water and ions, particularly phosphate and sulfate anions. To this end, gaining deeper insight into the molecular mechanisms of water, ion, and proton transport of the advanced materials is essential for guiding the development of next-generation proton-conducting materials.^19–22^

The Hofmeister effect, originally identified in the late 19th century, describes the specific influence of ions on the behavior of solutes in aqueous solutions, particularly their solubility, stability, and aggregation.^23–26^ This ion-specific effect is characterized by a distinct ion ranking, known as the Hofmeister series, which reflects the differential ability of ions to stabilize or destabilize solute structures through their interactions with water and other solutes. The Hofmeister series, typically studied for anions, is broadly divided into kosmotropes (structure makers), which enhance the water structure and salt out solutes, and chaotropes (structure breakers), which disrupt the water structure and salt in solutes (Figure 1a).^27–31^ While the Hofmeister effect has been extensively studied in biological, chemical, and physical aqueous systems,^30–32^ its systematic investigation in solid-state materials, particularly in relation to the Grotthuss-type proton-hopping mechanism, remains scarce.

**Figure 1:**
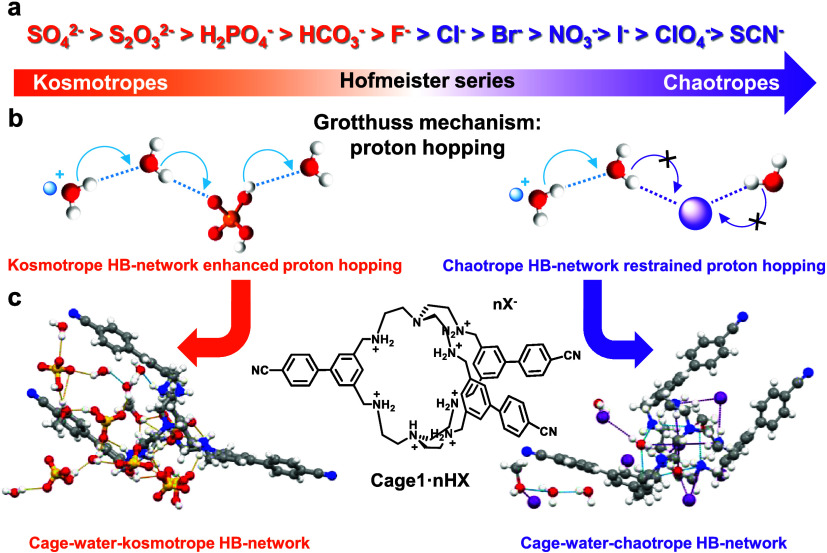
(a) Schematic diagram of the classical Hofmeister series from kosmotropes to chaotropes. (b) Schematic diagram of the Grotthuss mechanism showing kosmotrope HB-network-enhanced proton hopping and chaotrope HB-network-restrained proton hopping. (c) Chemical structure of the Cage1 series, its cage–water–kosmotrope HB-network for enhanced proton hopping, and cage–water–chaotrope HB-network-restrained proton hopping. Hydrogen bond color code (dashed line): orange, HSO_4_^–^-based O···H; cyan, O/N···H; purple, I^–^-based I^–^···H.

Organic cages with well-defined intrinsic cavities have attracted growing interest due to their unique combination of solution processability, chemical stability, and structural tunability.^33–41^ Unlike MOFs^42–44^ and COFs,^45–47^ they can readily self-assemble in solution into defect-free crystalline porous architectures, enabling precise control over proton transport pathways for proton-conducting applications.^16,48,49^ Herein, we report a series of organic cage salts exhibiting a pronounced Hofmeister effect on proton conduction. Kosmotropes, unlike their chaotropic counterparts, assemble cage–anion–water HB-network that substantially enhances proton conductivity (Figure 1b,c). Notably, kosmotropic Cage1-H_2_SO_4_ exhibits over 2 orders of magnitude enhancement compared with chaotropic Cage1-HI, achieving up to 1.03 × 10^–1^ S cm^–1^ at 333 K under 95% RH. Single-crystal analyses, together with MD results, reveal that kosmotropic anions immobilized within the cage framework create robust HB-network that induce efficient proton dynamics, thereby greatly facilitating proton transfer in these cage-based supramolecular conductors. This work provides molecular-level insights into the Hofmeister effect on proton conduction and establishes design principles for advanced proton-conducting materials.

## Results and Discussion

### Synthesis of Cage1·*n*HX Series

In order to study the Hofmeister effect in solids, a new triskelion organic cage, Cage1, with a polyaza cage skeleton and extended −CN functional groups, was designed and synthesized. The exo-functional −CN can not only act as a hydrogen bond acceptor but also help modulate the electronic environment for proton transfer through its strong dipole moment. More importantly, it serves as a rigid exo-functional group that extends the porous architecture and enhances the structural order. Together with its polyaza skeleton, Cage1 shows great potential for forming a three-dimensional micropore architecture with abundant hydrogen-bonding sites.

As shown in Scheme S1, following the Suzuki coupling, the CN-containing dialdehyde precursor underwent imine condensation with tris­(2-aminoethyl)­amine (TREN), followed by reduction to yield triskelion Cage1. Subsequent treatment with concentrated acid (HX) solutions quantitatively protonates Cage1 to form Cage1 series salts, including Cage1-H_2_SO_4_, Cage1-H_3_PO_4_, Cage1-HCl, Cage1-HBr, Cage1-HNO_3_, and Cage1-HI. Their chemical structures and purities were confirmed by ^1^H and ^13^C nuclear magnetic resonance (NMR) spectroscopy and high-resolution mass spectrometry (HRMS). Detailed characterizations are provided in the Supporting Information (Scheme S1 and Figures S1–S18).

The polyaza framework architecture permits the systematic incorporation of a wide range of anions through acidification reactions, providing a versatile platform for investigating the Hofmeister effect. Meanwhile, the −CN functional groups introduced at the periphery directed the self-assembly of the molecular cages into hierarchically ordered and structurally sophisticated architectures. Thus, unique micropore structures can be formed through the intrinsic cavity and extrinsic channels of the exo-functional organic cages, establishing a perfect porous architecture to confine various anions and water molecules for subsequent proton transfer applications.

### Hofmeister Effect in Cage1·*n*HX Series

T-shaped Teflon cells were assembled, with powder pellets sandwiched between two platinum foil blocking electrodes for the proton conductivity test. The homogeneous crystallinity and stability of the pellet samples are confirmed by FTIR, XPS, and PXRD (Figures S19–S21). We first measured the proton conductivities of the Cage1·*n*HX series using alternating current electrochemical impedance spectroscopy (EIS) across a temperature range of 293–333 K under a constant relative humidity (RH) of 95% (Figures 2a–c, S22–S24, and Table S1). All Cage1·*n*HX samples exhibit a positive correlation between proton conductivity and temperature, with the conductivity increasing as the temperature increases (Figure 2d). Notably, Cage1-H_2_SO_4_ and Cage1-H_3_PO_4_, both containing kosmotropic anions, consistently demonstrate the highest proton conductivities across the entire temperature range. Furthermore, at any given temperature, a clear decreasing trend in conductivity is observed across the Cage1 series in the following order: Cage1-H_2_SO_4_ > Cage1-H_3_PO_4_ > Cage1-HCl > Cage1-HBr > Cage1-HNO_3_ > Cage1-HI (Figure 2d). For instance, at 333 K and 95% RH, the proton conductivity progressively declines from Cage1-H_2_SO_4_ (1.03 × 10^–1^ S cm^–1^) to Cage1-HI (4.95 × 10^–4^ S cm^–1^) (Figure 2e), in line with the classical Hofmeister series (Figure 1). Remarkably, Cage1-H_2_SO_4_ attains a peak conductivity of 1.03 × 10^–1^ S cm^–1^ under these conditions, surpassing all reported supramolecular hosts, including both macrocycles and cages (Figure 2f),^15,16,48–50^ as well as the benchmark commercial Nafion 117 (7.5 × 10^–2^ S cm^–1^ at 333 K, 98% RH).^51^ The stability and reproducibility of the supramolecular conductor were further confirmed by long-term and temperature-dependent proton conductivity tests (Figure S25).

**Figure 2:**
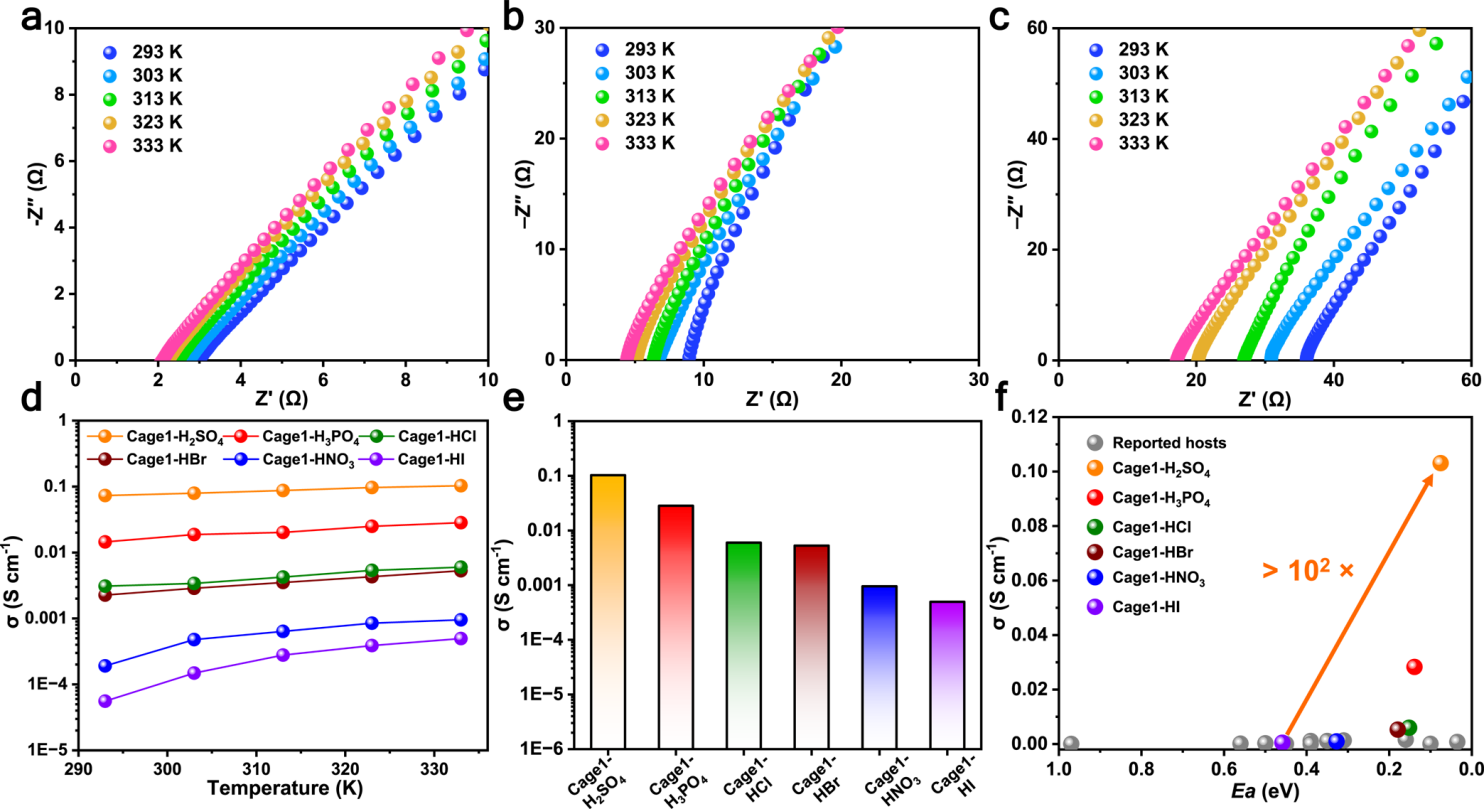
Nyquist plots of (a) Cage1-H_2_SO_4_, (b) Cage1-H_3_PO_4_, and (c) Cage1-HCl as a function of temperature (293–333 K) under 95% RH. (d) Proton conductivity of Cage1 series under 95% RH at different temperatures. (e) Proton conductivity of the Cage1 series at 333 K under 95% RH. (f) Comparison of proton conductivity and activation energy (*E*_a_) of Cage1·*n*HX series and other reported supramolecular hosts. Detailed data are provided in Table S3.

As noted above, water molecules play a pivotal role in the kosmotrope/chaotrope framework of the Hofmeister effects. We therefore examined the proton conductivity of the Cage1 series under varying relative humidity (Table S2, and Figures S26–S31). As shown in Figure 3a, all the Cage1 salts exhibit enhanced conductivity with increasing RH, reaching the highest proton conductivity value at 95% RH. Moreover, at all RH levels, Cage1 series consistently follows a clear Hofmeister trend, decreasing from Cage1-H_2_SO_4_ to Cage1-HI. To gain deeper insight, we compared the water uptake capacities of the two extreme cases, Cage1-H_2_SO_4_ and Cage1-HI. The water vapor adsorption isotherms measured at 298 K (Figure 3b) show a substantially higher water uptake for Cage1-H_2_SO_4_ (19.4 mmol g^–1^) than for Cage1-HI (12.8 mmol g^–1^). Across the entire Cage1 series, water uptake generally follows the Hofmeister series, with Cage1-H_2_SO_4_ and Cage1-H_3_PO_4_ showing the highest values (Figure S32). The enhanced water adsorption behavior of the kosmotropic salts is further supported by TGA analysis (Figure S33). These findings indicate that introducing structure-making kosmotropes, such as sulfates and phosphates, markedly enhances water adsorption, thereby potentially improving the performance of supramolecular proton conductors.

**Figure 3:**
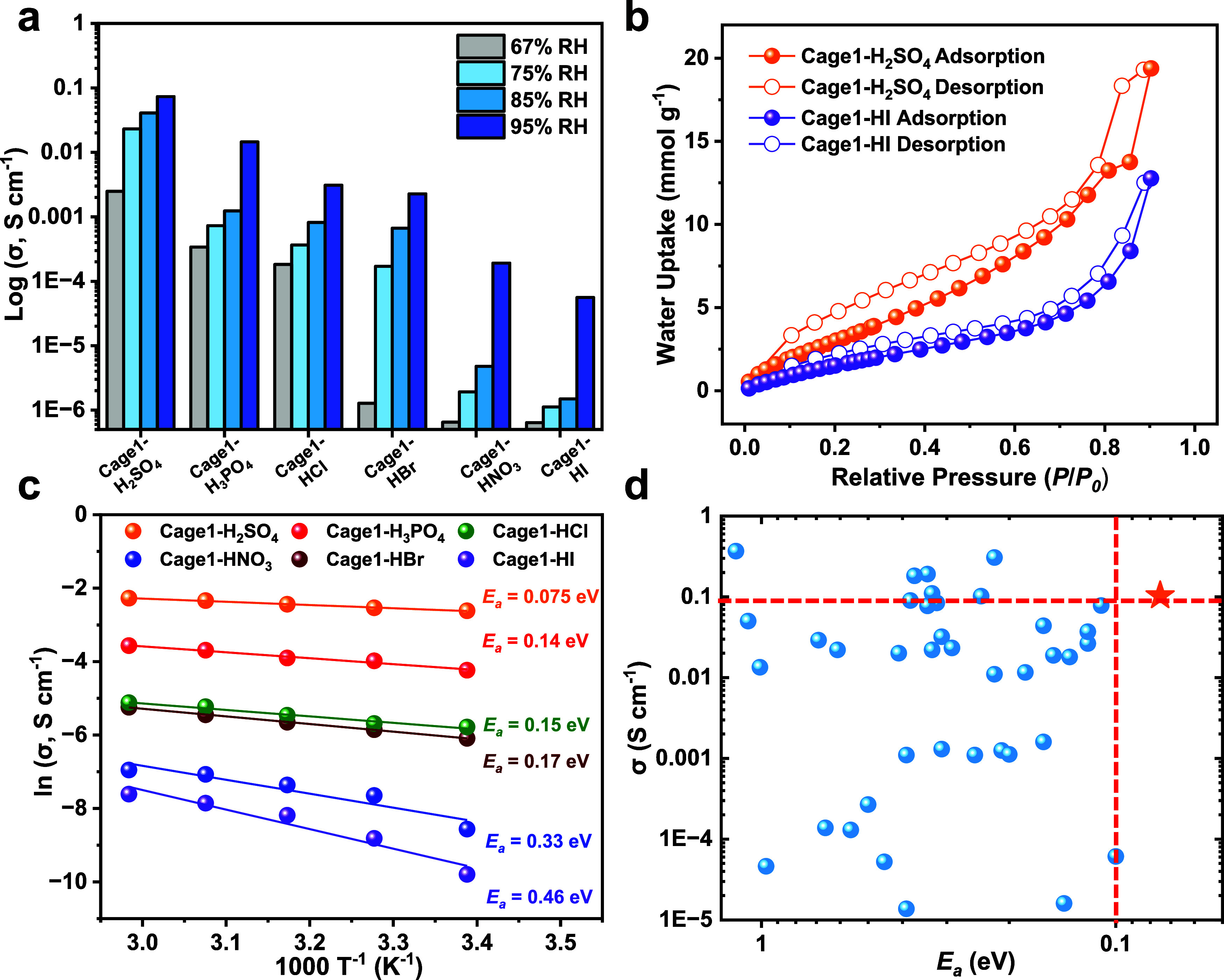
(a) Proton conductivity of the Cage1 series at 293 K under 67%–95% RH. (b) Water vapor adsorption/desorption isotherms of Cage1-H_2_SO_4_ and Cage1-HI. (c) Arrhenius plots of the proton conductivity of the Cage1 series at 95% RH with their calculated activation energies (*E*_a_). (d) Comparison of proton conductivity vs *E*_a_ of Cage1-H_2_SO_4_ and other reported proton-conducting materials. Detailed data are provided in Table S4.

The clear Hofmeister effect was also reflected in the activation energies (*E*_a_), obtained by least-squares fitting of Arrhenius plots from temperature-dependent conductivities at 95% RH (Figure 3c). Proton conduction generally proceeds via two mechanisms: the efficient Grotthuss hopping mechanism (*E*_a_ < 0.4 eV), which involves proton transfer through structural diffusion of excess protons (protonic charge defects) along a HB-network, and the vehicular mechanism (*E*_a_ > 0.4 eV), where proton transport occurs via the diffusion of protonated species.^52–54^ The *E*_a_ values of the Cage1 series follow the Hofmeister trend, indicating predominant Grotthuss-type proton transport, with the exception of the most chaotropic system, Cage1-HI (*E*_a_ = 0.46 eV). Notably, kosmotropic Cage1-H_2_SO_4_ (0.075 eV) and Cage1-H_3_PO_4_ (0.14 eV) show the lowest *E*_a_ values in the series, indicating a more stable and efficient proton-hopping efficacy. Combining ultrahigh proton conductivity with an exceptionally low *E*_a_, Cage1-H_2_SO_4_ outperforms most state-of-the-art proton-conducting materials (Figure 3d). These results underscore that, despite the similar protonated porous frameworks with abundant hydrogen-bonding sites, the counteranions play a decisive role in enabling rapid proton transport. In particular, kosmotropic anions markedly enhance the proton conductivity of supramolecular conductors.

### Mechanism Exploration at the Molecular Level

To further explore the underlying molecular-level mechanism, we attempted to crystallize the cage materials to reveal their atomic-scale structural details. After numerous attempts, crystals of Cage1-H_2_SO_4_, Cage1-H_3_PO_4_, Cage1-HCl, and Cage1-HI are successfully obtained.^37^ Considering that Cage1-H_2_SO_4_ and Cage1-H_3_PO_4_ possess classical kosmotropic anions, while Cage1-HCl and Cage1-HI have chloride and iodide as the halogen element and chaotrope, respectively, these crystals can serve as a good comparison for analyzing and understanding the mechanism (Tables S5–S8, Figures 4, and S34–S43).

**Figure 4:**
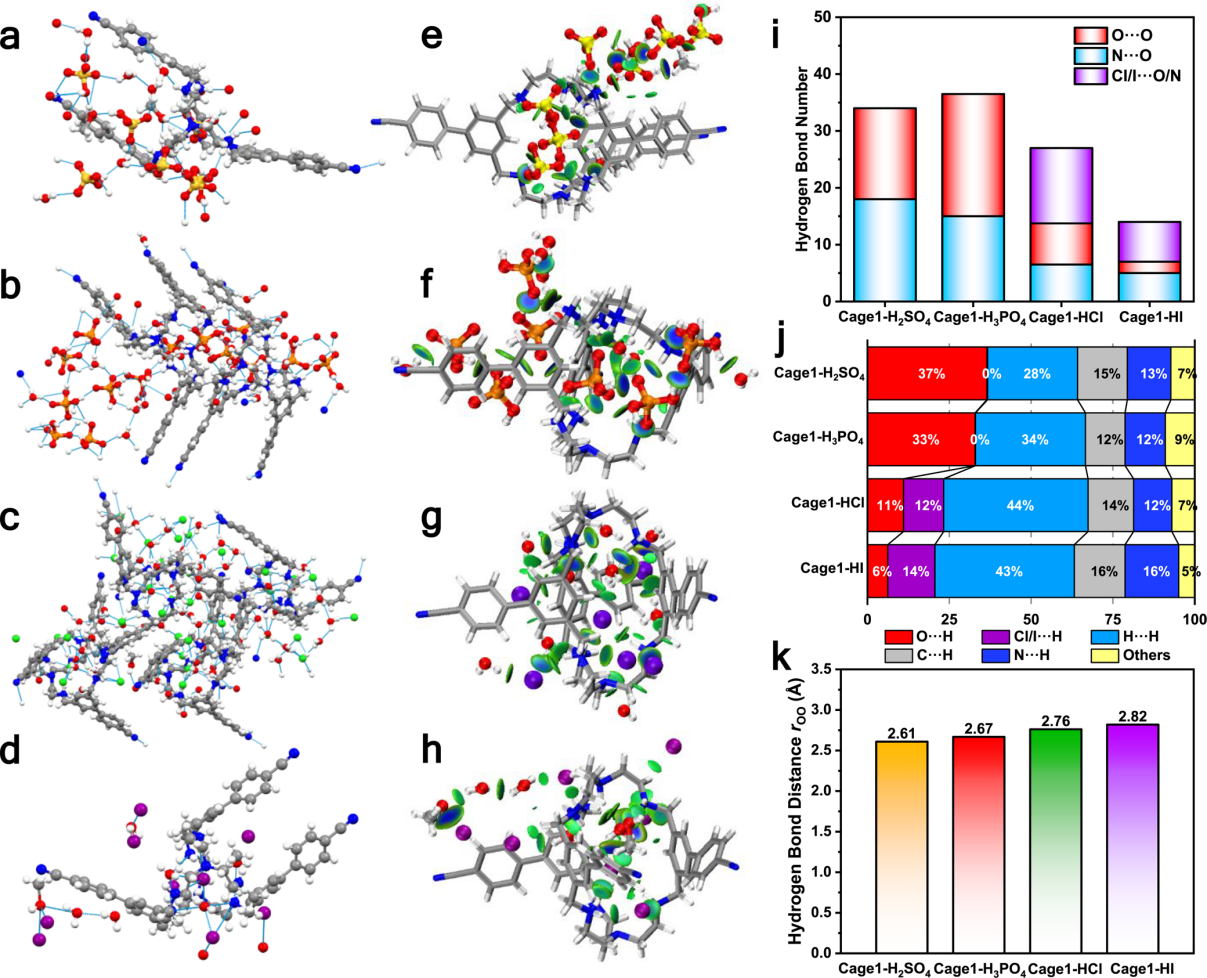
Crystal structures of (a) Cage1-H_2_SO_4_, (b) Cage1-H_3_PO_4_, (c) Cage1-HCl, and (d) Cage1-HI showing their HB-network. Element color code: Red, O; blue, N; gray, C; white, H; yellow, S; orange, P; green, Cl; purple, I. IGMH maps of (e) Cage1-H_2_SO_4_, (f) Cage1-H_3_PO_4_, (g) Cage1-HCl, and (h) Cage1-HI, highlighting various supramolecular interactions (green region), especially hydrogen bonding (blue region). *Sign*(λ_2_)­ρ*-*colored isosurfaces of *δg* ^inter^ = 0.01 au, corresponding to the IGMH analyses. The cage is shown in stick mode, and the other species are shown in ball–stick mode. Element color code: Red, O; blue, N; gray, C; white, H; yellow, S; orange, P; violet, Cl; purple, I. (i) Hydrogen bond number analysis, (j) relative contributions of various interactions to the Hirshfeld surface area, and (k) averaged O···O hydrogen bond distances (*r*_OO_) in Cage1-H_2_SO_4_, Cage1-H_3_PO_4_, Cage1-HCl, and Cage1-HI crystals.

As shown in Figure 4a, in the Cage1-H_2_SO_4_ crystal, there are 7 HSO_4_^–^ ions surrounding each distorted heptaprotonated cage, with one located inside the cage cavity and the remaining six in the extrinsic cavity, collectively forming a robust three-dimensional HB-network with the cage and surrounding water molecules (Figures S38a and S39). Most hydrogen-bonding sites act as bridges, interacting with at least two other sites. Notably, the HSO_4_^–^ can even serve as multiple hydrogen-bonding sites that can simultaneously form more than 4 hydrogen bonds with surrounding HSO_4_^–^, –NH_2_^+^, or H_2_O molecules (Figures 4a and S39). The average hydrogen bond number analysis (Figure 4i) shows that there are around 34 hydrogen bonds (16 OH···O and 18 OH···N/NH···O hydrogen bonds, Table S9) per protonated cage and 2.8 hydrogen bonds per water molecule (Figure S46). The calculated Hirshfeld surface analysis, which provides a quantitative representation of the contribution of the supramolecular interactions within the host–guest crystal system, further suggests that hydrogen bonding (O···H) contributed by HSO_4_^–^ and water plays a dominant role in Cage1-H_2_SO_4_ (37%) among the various supramolecular interactions in this Cage1-H_2_SO_4_ (Figures 4j and S43a). Similar situations are seen in the Cage1-H_3_PO_4_ systems (Figures 4b, S38b, and S40). The kosmotrope H_2_PO_4_^–^ can also simultaneously form 4–8 hydrogen bonds to realize around 36 hydrogen bonds per cage, 2.8 hydrogen bonds per water molecule, and 33% contributions to the various intermolecular contributions (Figures 4i,j, S43b, S46, and Table S10). Collectively, abundant hydrogen bonds are formed in the crystals of kosmotropic Cage1-H_2_SO_4_ and Cage1-H_3_PO_4_, with the kosmotropes themselves functioning as proton-hopping sites, facilitating the formation of extended 3D Grotthuss networks for efficient proton conduction.

In Cage1-HCl (Figures 4c, S38c, and S41) and Cage1-HI (Figures 4d, S38d, and S42) systems, the hydrogen bond numbers obviously decrease. As shown in Figure 4i, around 27 hydrogen bonds per cage are found in the Cage1-HCl crystal. Moreover, the Cl^–^ involving hydrogen bonds disrupt the proton-hopping process, as chloride or iodide itself is unable to conduct protons but basically serves as a poor hydrogen acceptor and potential proton terminator. In this regard, only 13.75 efficient hydrogen bonds (7.25 OH···O and 6.5 OH···N/NH···O), together with inefficient 13.25 OH···Cl^–^/NH···Cl^–^ interactions, are formed per protonated cage in Cage1-HCl (Figure 4i, and Table S11). In the analysis of hydrogen bonds per water molecule, only 2.2 are found with 0.6 inefficient OH···Cl^–^ interactions (Figure S46). In Cage-HI, the hydrogen bond number even drops to 14: there are only 7 efficient hydrogen bonds accompanied by 7 inefficient I^–^-related ones (Figure 4i, and Table S12). Only 1.5 hydrogen bonds are found per water molecule, including 0.5 inefficient I^–^-related ones (Figure S46). The lack of hydrogen bonds results in a discontinuous HB-network (Figures 4d and S42), which hinders proton hopping, thereby favoring less efficient, vehicular-type proton transport in Cage1-HI. This is also supported by the calculated Hirshfeld surface analysis. As shown in Figure 4j, the Cl^–^-associated hydrogen bonds (12%) contribute more than the water-related (11%) hydrogen bonds. In the Cage1-HI crystal, the I^–^-associated hydrogen bond contribution even increases to 14%, while the water-related one drops dramatically to 6% (Figure S43c,d).

An independent gradient model based on Hirshfeld partitioning (IGMH) analysis was performed to visualize and compare these intermolecular interactions.^55^ As shown in Figure 4e,f, IGMH maps of the kosmotropic Cage1-H_2_SO_4_ and Cage1-H_3_PO_4_ complexes reveal various noncovalent interactions (shown in green), particularly the multiple hydrogen-bonding interactions (highlighted in blue) among HSO_4_^–^/H_2_PO_4_^–^ and –NH_2_^+^ within the cage as well as H_2_O (Figures S44a,b). In the chaotropic Cage1-HCl and Cage1-HI, chloride and iodide anions compete to form hydrogen bonds with –NH_2_^+^ and H_2_O donors through various OH···Cl^–^/I^–^ and N–H···Cl^–^/I^–^ interactions (Figures 4g,h, and S44c,d). These weaker interactions, indicated by the greener or lighter blue regions, reduce the overall strength of the chaotropic HB-network, thereby hindering efficient proton hopping.

To enable a quantitative analysis and robust comparison of the HB-network, the hydrogen bond distances were calculated. HSO_4_^–^ and H_2_PO_4_^–^ anions readily form stronger hydrogen bonds with water molecules and the –NH groups of the cage, as evidenced by their shorter intermolecular hydrogen bonds (O···O distances between adjacent molecules, *r*_OO_, shorter than 2.85 Å in water). As shown in Figure 4k, the average *r*_OO_ values in Cage1-H_2_SO_4_ and Cage1-H_3_PO_4_ are only 2.61 Å and 2.67 Å, respectively. These values are significantly shorter than the 2.76 Å observed in Cage1-HCl and the 2.82 Å observed in Cage1-HI (Tables S9–S12). When analyzing the average hydrogen bond distance of all donor–acceptor pairs, Cage1-H_2_SO_4_ (2.71 Å) and Cage1-H_3_PO_4_ (2.71 Å) still show a considerably smaller *r*_*xy*_ value than Cage1-HCl (2.92 Å) and Cage1-HI (2.98 Å, Figure S45). These notably shorter hydrogen bonds in the kosmotropic Cage1-H_2_SO_4_ and Cage1-H_3_PO_4_ facilitate a stronger HB-network, enabling more efficient proton hopping. These findings, showing an increasing trend in the hydrogen bond distance from HSO_4_^–^ to I^–^, further suggest that kosmotropic anions can not only form much more hydrogen bonds but also form much stronger hydrogen bonds and HB-network than chaotropic anions.

Despite minor disorder and refinement alerts resulting from weak diffraction, the large asymmetric unit and overall data quality sufficiently capture the cage–anion–water HB-network, providing strong proof of the mechanistic analysis. Ab initio molecular dynamics (AIMD) simulations were further carried out using the CP2K package to investigate the dynamic hydrogen-bonding behavior.^56,57^ The hydrogen bond numbers and radial distribution functions, *g­(r)*, of water/Cage1/anion were calculated and analyzed. We first calculated and compared the *g*_oo_ *(r)*, representing just the oxygen–oxygen radial distribution in the system (Figure S47). The *g*_oo_ *(r)* of Cage1-H_2_SO_4_ and Cage1-H_3_PO_4_ shows strong peaks at 2.47 Å and 2.56 Å, respectively. Weak and broad peaks at around 2.74 Å and 2.83 Å are observed for Cage1-HCl and Cage1-HI, respectively. Considering the whole HB-network system, the *g­(r)* function provides a broader insight into the local structure and molecular organization of water molecules, protonated cages, and anions. As shown in Figure S48, although Cage1-HCl and Cage1-HI still display weak and broad peaks, their peak values increase dramatically to around 3.03 Å and 3.26 Å, respectively, which primarily arise from the Cl^–^ and I^–^-associated weaker hydrogen bonds with longer bond lengths. By contrast, *g­(r)* in Cage1-H_2_SO_4_ and Cage1-H_3_PO_4_ still exhibits pronounced peaks at 2.47 Å and 2.56 Å, respectively, indicating strong dynamic hydrogen-bonding interactions among HSO_4_^–^ or H_2_PO_4_^–^ anions, cages, and water molecules. Besides, the simulated dynamic hydrogen bond number also decreases from Cage1-H_2_SO_4_, Cage1-H_3_PO_4_, Cage1-HCl to Cage1-HI (Figure S49), which is in good agreement with the crystal analysis results. Collectively, the MD simulations confirm that kosmotropic anions more readily form stronger hydrogen bonds and HB-network than chaotropic anions, establishing cage–kosmotrope–water ternary hydrogen bond systems that enable highly efficient proton conduction.

## Conclusions

Although the Hofmeister effect has been extensively explored in solution-phase solvation science, and Grotthuss-type proton hopping is well established in solid-state proton conductors, the mechanistic connection between these two phenomena has remained underexplored to date. Here, we demonstrate the manifestation of the Hofmeister effect in the solid state through a series of cage-based supramolecular conductors exhibiting ion-specific proton conductivity consistent with Hofmeister ordering. Switching from chaotropic HI to kosmotropic H_2_SO_4_ boosts Cage1 proton conductivity by over 2 orders of magnitude, reaching 1.03 × 10^–1^ S cm^–1^ at 333 K and 95% RH. Systematic investigation reveals that kosmotropic anions markedly enhance proton transport by stabilizing extended HB-network and participating as potential active hopping sites, in contrast to chaotropic anions, which, despite forming hydrogen bonds, do not support efficient proton transfer. Single-crystal structural and MD analyses uncover the molecular basis of this enhancement, showing that ion-specific proton transfer is mediated by distinct cage–anion–water hydrogen-bonding motifs. The spatial confinement and stabilization of kosmotropes and water molecules within the supramolecular cage framework facilitate efficient Grotthuss-type proton hopping. To the best of our knowledge, this is the first demonstration of kosmotrope-promoted proton conduction in solid-state materials governed by the Hofmeister effect. These findings not only establish a previously unexplored link between ion-specific effects and proton dynamics in the solid state but also provide a design paradigm for next-generation supramolecular materials with high proton conductivity.

## Supplementary Material



## Notes

Detailed materials and methods; synthesis and characterization details, including NMR spectra, proton conductivity, and other characterization data; single-crystal X-ray diffraction and analysis; calculations and simulations (PDF)
