# Supplementary material for: Kosmotrope-Promoted Proton Hopping in Supramolecular Conductors
Source: J Am Chem Soc. 2026 May 14;148(24):24942–9. doi: 10.1021/jacs.6c03867 (PMC13307352; doi:10.1021/jacs.6c03867)
Supplement: Supplementary file 1 [file ja6c03867_si_001.pdf]

Supporting Information for

Kosmotrope-Promoted Proton Hopping in Supramolecular Conductors

Wei-Bin Lin,[1] Yongjiu Lei,[2] Pei Yu,[1] Lukman O. Alimi,[1] Jinrong Wang,[1] Basem A. Moosa,[1] Niveen M. Khashab[1]*

[1] Smart Hybrid Materials Laboratory (SHMs), Physical Science and Engineering Division, King Abdullah University of Science and Technology (KAUST), Thuwal 23955-6900, Saudi Arabia.

[2] Materials Science and Engineering, Physical Science and Engineering Division, King Abdullah University of Science and Technology (KAUST), Thuwal 23955-6900, Saudi Arabia.

*Niveen M. Khashab. Email niveen.khashab@kaust.edu.sa

Correspondence to: niveen.khashab@kaust.edu.sa

Table of contents:

Materials and Methods

Synthesis and Characterization

Single Crystal X-ray Diffraction and Analysis

Calculations and Simulations

References

Materials and Methods

Materials

All commercial reagents were obtained from Sigma-Aldrich and used without further purification. Compound **3** was prepared according to a previous report.[1] Flash column chromatography was performed on 200-300 mesh silica gel. Thin-layer chromatography (TLC) was performed on precoated silica gel aluminum plates and observed under UV light.

Instruments and characterization

^{1}H and ^{13}C nuclear magnetic resonance (NMR) spectra were recorded on Brucker® AVIII 400 MHz NMR spectrometers. High-resolution mass spectra (HRMS) were determined in the MALDI-TOF ESI mode. Single crystal data was collected on a Bruker Smart APEXII CCD diffractometer using graphite monochromated Ga Kα or Ga Kα radiation. The water uptake experiment was performed by using Micromeritics ASAP 2020 and ASAP 2420 surface area and pore size analyzer. Thermogravimetric analyses (TGA) were performed using a TGA-TA Discovery 5500 instrument, under an air atmosphere from 0 to 800 °C, with a heating rate of 10 °C min^{-1}. Fourier-transform infrared spectroscopy (FT-IR) spectra were recorded on a Perkin-Elmer Paragon 1000 spectrometer at frequencies ranging from 4000 to 500 cm^{-1} at room temperature. Powder X-ray diffraction (PXRD) analyses were performed using a Bruker D8 Advance X-ray diffractometer at room temperature. The X-ray photoelectron spectroscopy (XPS) analyses were performed on the Kartos Amicus system to identify the surface element composition.

The proton conductivity was measured with an EC Labs Bio-Logic (SP-300) potentiostat using banana plug cables. T-shaped Teflon cells were assembled, with the pellets sandwiched between two platinum foil blocking electrodes. The 2 probe (quasi four probe) electrochemical impedance spectroscopy was measured using a sinusoidal amplitude of 10.0 mV over the frequency range 1 MHz–20 mHz. Proton conductivity measurements at different temperatures were conducted using the standard environmental chamber: SDH01 Temperature & Humidity Chamber.

Synthesis and Characterization

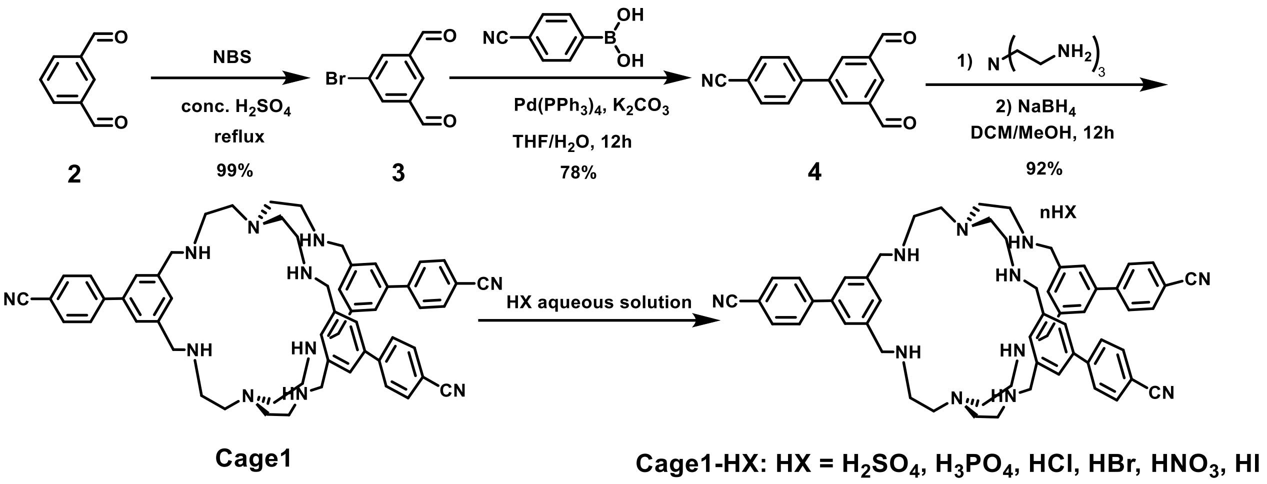

Scheme S1. Synthesis of the organic cage salts Cage1-HX.

Synthesis of 4: To a mixture of **3** (2.12 g, 10 mmol), 4-cyanophenylboronic acid (1.76 g, 12 mmol), and K_2CO_3 (5.52 g, 40 mmol) dissolved in tetrahydrofuran (THF, 80 mL) and water (40 mL) under an argon atmosphere was added a catalytic amount of $Pd(PPh_3)_4$ (0.578 g, 5% mol). The resulting mixture was stirred for 12 hours at 80°C under an argon atmosphere, cooled to room temperature, and then evaporated to remove the THF and other volatiles. Dichloromethane (DCM, 50 mL) and water (20 mL) were added to the residue. After mixing, the organic layer was washed with water twice (2 × 50 mL), dried over anhydrous sodium sulfate, and then concentrated *in vacuo*. The resulting residue was subjected to silica gel column chromatography (eluent: DCM/hexane) to afford **4** as a yellowish solid (78%). ^{1}H NMR ($CDCl_3$, 400 MHz): δ = 10.20 (s, 2H), 8.42 (s, 1H), 8.37 (s, 2H), 7.83 (d, *J* = 8.4 Hz, 2H), 7.79 (d, *J* = 8.6 Hz, 2H) ppm. ^{13}C NMR ($CDCl_3$, 100 MHz): δ = 190.68, 142.85, 141.48, 137.98, 133.23, 133.19, 132.86, 131.24, 130.76, 128.06 ppm. MALDI-TOF-HRMS (ESI): *m/z* 236.0706 [M+H]+; found: 236.0707.

Synthesis of Cage1: A mixture of aldehyde **4** (1.41 g, 6 mmol) and tris(2-aminoethyl)amine (0.585 g, 4 mmol) in 100 mL acetonitrile was stirred at room temperature for 24 h. The yellowish precipitate was isolated, washed with acetonitrile, and dried in vacuo to afford the yellowish imine product. Then sodium borohydride (0.38 g, 10 mmol) was added to the solution of imine product (0.89 g, 1.0 mmol) in DCM/MeOH (*v:v* = 2:1, 60 mL) at 0 °C. After the mixture was stirred for 6 hours at room temperature, water was added, and the residue was extracted with DCM and washed with aqueous sodium carbonate (5%). The organic solution was dried over sodium sulfate, and the

solvent was removed by rotary evaporation to give the organic cage Cage1 (92%). ^{1}H NMR ($CDCl_3$, 400 MHz): δ = 7.67 (s, 6H), 7.63 (d, J = 8.1 Hz, 6H), 7.39 (s, 6H), 7.16 (s, 3H), 3.68 (s, 12H), 2.71 (s, 12H), 2.64 (s, 12H) ppm. ^{13}C NMR ($CDCl_3$, 100 MHz): δ = 145.09, 139.30, 132.78, 132.70, 127.80, 127.67, 126.39, 118.98, 111.38, 53.80, 53.57, 47.71 ppm. MALDI-TOF-HRMS (ESI): m/z 902.5346 $[M+H]^+$; found: 902.5378.

Synthesis of the Cage1-H_2SO_4: To a solution of organic cage Cage1 (0.902 g, 1.0 mmol) was added dropwise an excess of conc. H_2SO_4 until the pH reached ~0. The resulting precipitate was collected and dried in vacuo to afford the corresponding white powder Cage1-H_2SO_4 (98%). ^{1}H NMR (DMSO-*d6*, 400 MHz): δ = 8.99 (s, 12H), 8.17 (s, 3H), 7.96 (d, *J* = 4.1 Hz, 6H), 7.95 (s, 6H), 7.92 (d, *J* = 4.3 Hz, 6H), 4.22 (s, 12H), 3.24 (s, 12H), 2.79 (s, 12H) ppm. ^{13}C NMR (DMSO-*d6*, 100 MHz): δ = 143.51, 138.38, 133.28, 133.05, 131.63, 129.27, 127.60, 118.78, 110.54, 50.84, 50.43, 46.06 ppm. MALDI-TOF-HRMS (ESI): m/z 451.7707 $[M-nH_2SO_4+2H]^{2+}$; found: 451.7720.

Synthesis of the Cage1-H_3PO_4: To a solution of organic cage Cage1 (0.902 g, 1.0 mmol) was added dropwise an excess of conc. H_3PO_4 until the pH reached ~0. The resulting precipitate was collected and dried in vacuo to afford the corresponding white powder Cage1-H_3PO_4 (99%). ^{1}H NMR (D_2O, 400 MHz): δ = 7.71 (s, 6H), 7.68 (d, *J* = 8.6 Hz, 6H), 7.65 (d, *J* = 6.5 Hz, 6H), 7.55 (s, 3H), 4.19 (s, 12H), 3.30 (s, 12H), 2.87 (s, 12H) ppm. ^{13}C NMR (D_2O, 100 MHz): δ = 143.36, 139.98, 132.97, 132.51, 130.85, 129.15, 127.38, 119.42, 110.22, 51.22, 50.51, 45.67 ppm. MALDI-TOF-HRMS (ESI): m/z 1000.5110 $[M-6H_3PO_4+H]^+$; found: 1000.4997.

Synthesis of Cage1-HCl: To a solution of organic cage Cage1 (0.902 g, 1.0 mmol) was added dropwise an excess of conc. HCl solution until the pH reached ~0. The resulting precipitate was collected and dried in vacuo to afford the corresponding white powder Cage1-HCl (98%). ^{1}H NMR (DMSO-*d6*, 400 MHz): δ = 9.48 (s, 12H), 8.10 (s, 6H), 8.03 (d, *J* = 8.5 Hz, 6H), 7.91 (d, *J* = 8.3 Hz, 6H), 7.62 (s, 3H), 4.18 (s, 12H), 3.19 (s, 12H), 2.88 (s, 12H) ppm. ^{13}C NMR (DMSO-*d6*, 100 MHz): δ = 143.56, 138.50, 133.14, 132.89, 132.25, 129.65, 127.82, 118.99, 110.69, 50.13, 50.01, 44.47 ppm. MALDI-TOF-HRMS (ESI): m/z 902.5341 $[M-6HCl+H]^+$; found: 902.5358.

Synthesis of Cage1-HBr: To a solution of organic cage Cage1 (0.902 g, 1.0 mmol) was added dropwise an excess of conc. HBr until the pH reached ~0. The resulting precipitate was collected and dried in vacuo to afford the corresponding white powder Cage1-HBr (96%). ^{1}H NMR (DMSO-*d6*, 400 MHz): δ = 8.99 (s, 12H), 8.09 (s, 6H), 8.02 (d, *J* = 8.5 Hz, 6H), 7.99 (d, *J* = 5.9 Hz, 6H), 7.69 (s, 3H), 4.27 (s, 12H), 3.24 (s, 12H), 2.85 (s, 12H) ppm. ^{13}C NMR (DMSO-*d6*, 100 MHz): δ

= 143.27, 138.35, 133.03, 132.48, 129.72, 127.56, 126.93, 118.71, 110.65, 50.32, 49.84, 44.60 ppm. MALDI-TOF-HRMS (ESI): m/z 451.7707 $[M\text{-}nHBr+2H]^{2+}$; found: 451.7660.

Synthesis of Cage1-HNO₃: To a solution of organic cage Cage1 (0.902 g, 1.0 mmol) was added dropwise an excess of conc. HNO_3 solution until the pH reached ~0. The resulting precipitate was collected and dried in vacuo to afford the corresponding white powder Cage1-HNO_3 (98%). ^1H NMR (DMSO-d_6, 400 MHz): δ = 8.77 (s, 12H), 8.02 (d, *J* = 8.5 Hz, 6H), 8.01 (s, 6H), 7.93 (d, *J* = 8.3 Hz, 6H), 7.54 (s, 3H), 4.24 (s, 12H), 3.13 (s, 12H), 2.76 (s, 12H) ppm. ^{13}C NMR (DMSO-d_6, 100 MHz): δ = 144.58, 138.77, 133.19, 132.95, 129.47, 127.43, 118.70, 112.98, 110.81, 50.49, 50.01, 44.66 ppm. MALDI-TOF-HRMS (ESI): m/z 451.7707 $[M\text{-}nHNO_3+2H]^{2+}$; found: 451.7675.

Synthesis of Cage1-HI: To a solution of organic cage Cage1 (0.902 g, 1.0 mmol) was added dropwise an excess of conc. HBr solution until the pH reached ~0. The resulting precipitate was collected and dried in vacuo to afford the corresponding yellow powder Cage1-HI (97%). ^1H NMR (DMSO-d_6, 400 MHz): δ = 8.81 (s, 12H), 8.05 (d, *J* = 5.2 Hz, 6H), 8.04 (s, 6H), 7.94 (d, *J* = 8.2 Hz, 6H), 7.73 (s, 3H), 4.30 (s, 12H), 3.23 (s, 12H), 2.80 (s, 12H) ppm. ^{13}C NMR (DMSO-d_6, 100 MHz): δ = 143.14, 138.61, 133.14, 132.71, 129.55, 127.54, 127.43, 118.65, 110.76, 50.38, 49.96, 44.86 ppm. MALDI-TOF-HRMS (ESI): m/z 451.7707 $[M\text{-}nHI+2H]^{2+}$; found: 451.7667.

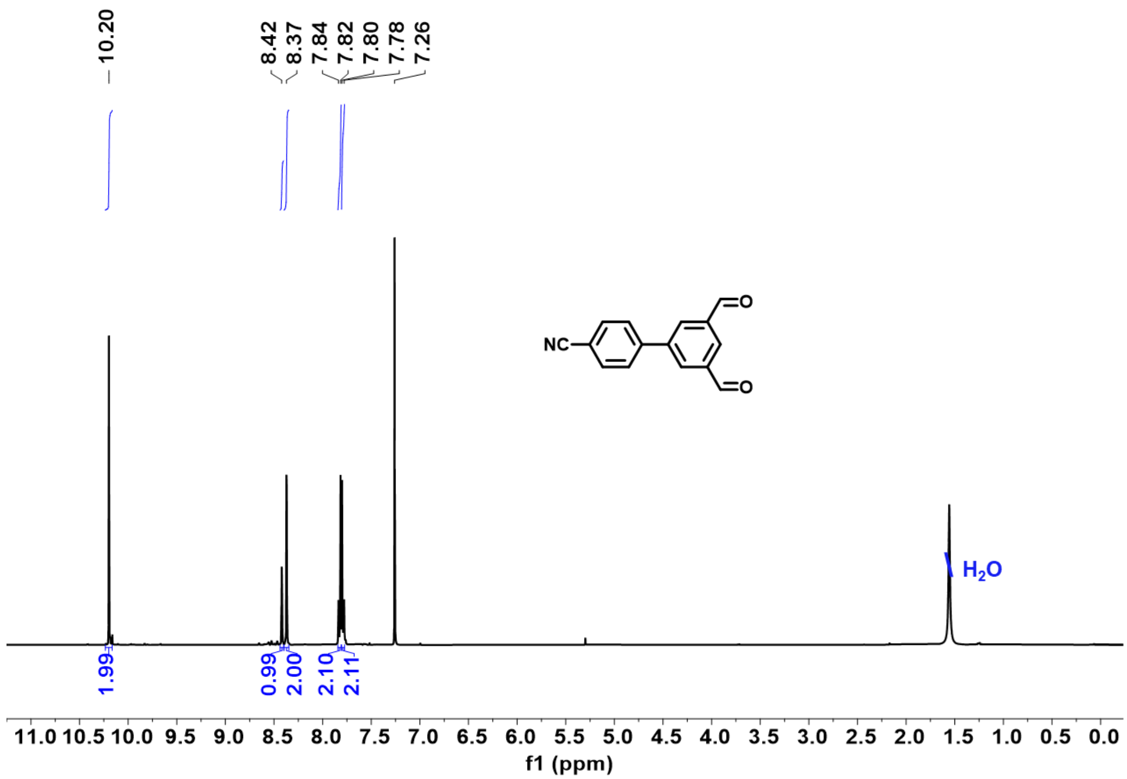

Figure S1. ^{1}H NMR spectrum of **4** in solution (400 MHz, $CDCl_3$, 25 °C).

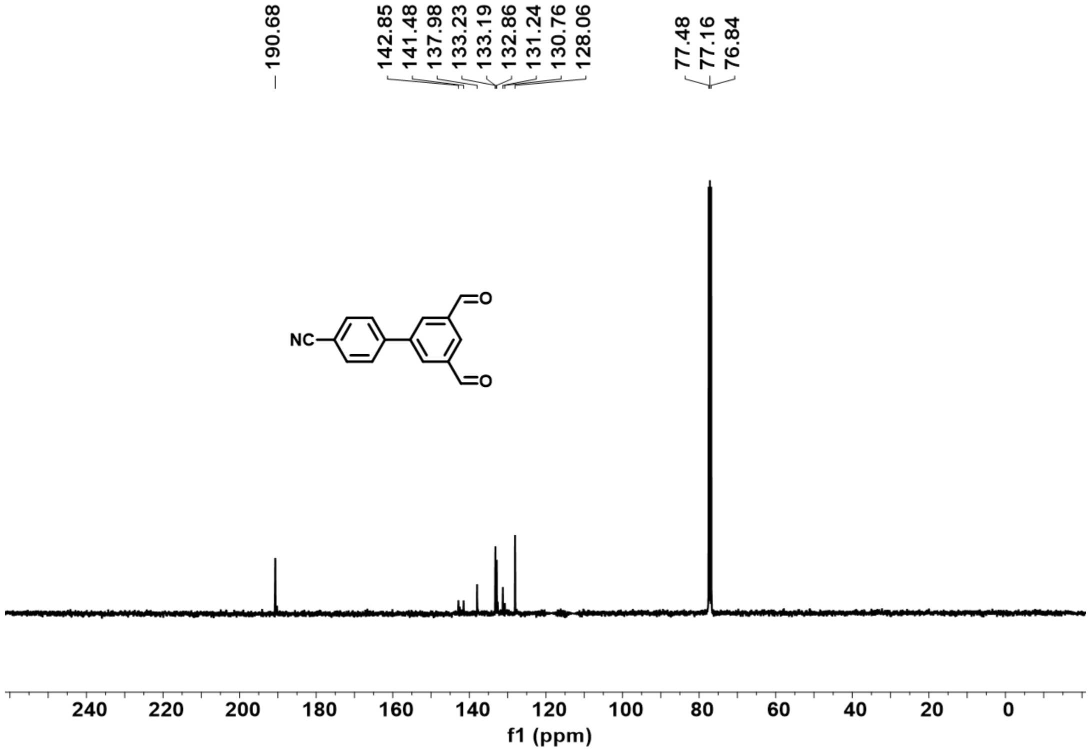

Figure S2. ^{13}C NMR spectrum of **4** in solution (100 MHz, $CDCl_3$, 25 °C).

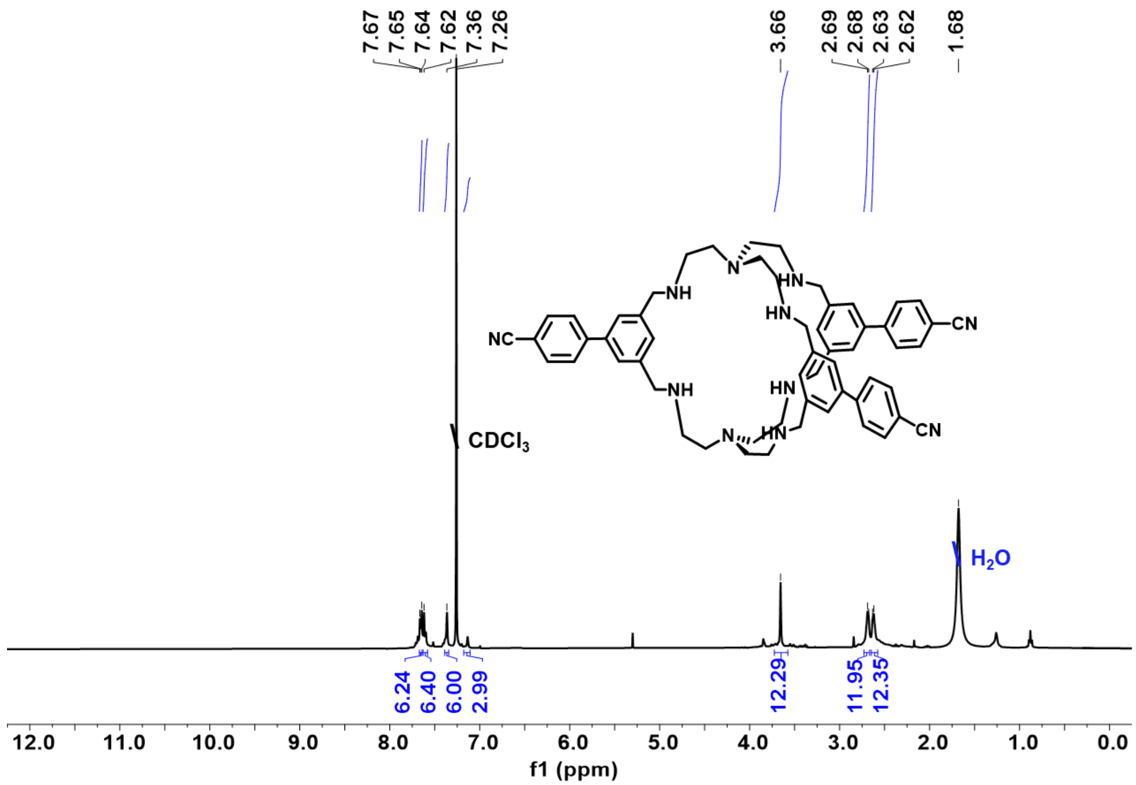

Figure S3. ^{1}H NMR spectrum of Cage1 in solution (400 MHz, $CDCl_3$, 25 °C).

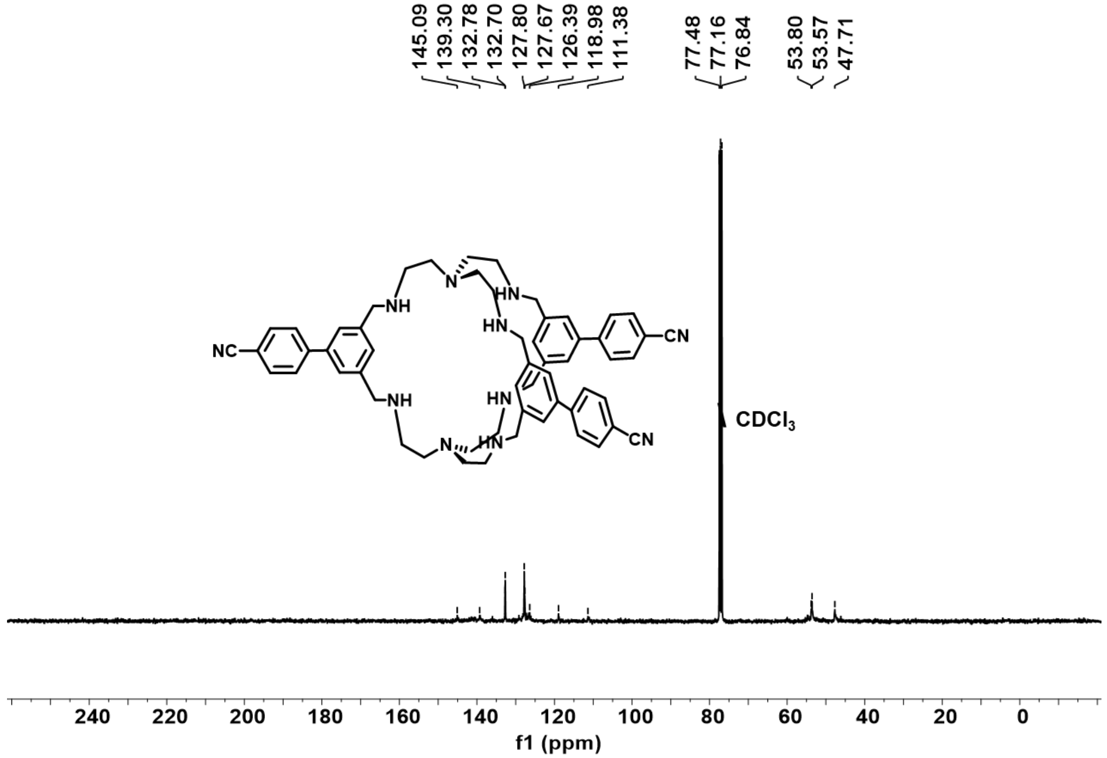

Figure S4. ^{13}C NMR spectrum of Cage1 in solution (100 MHz, $CDCl_3$, 25 °C).

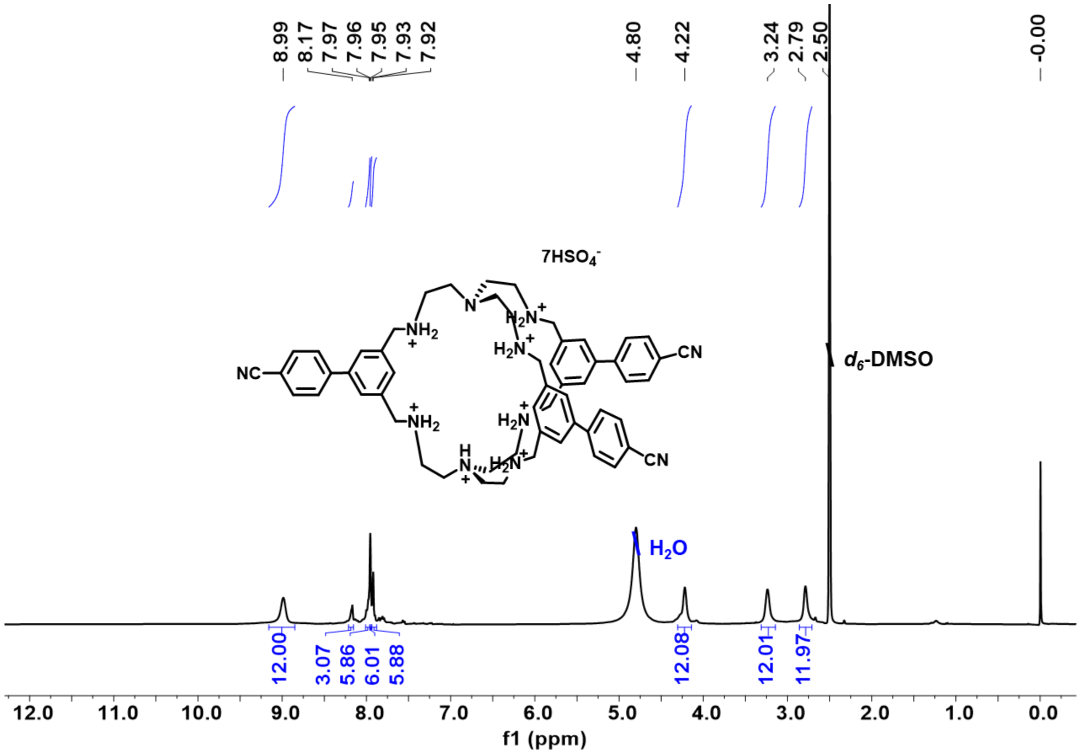

Figure S5. ^{1}H NMR spectrum of Cage1-H_2SO_4 in solution (400 MHz, DMSO-d_6, 25 °C).

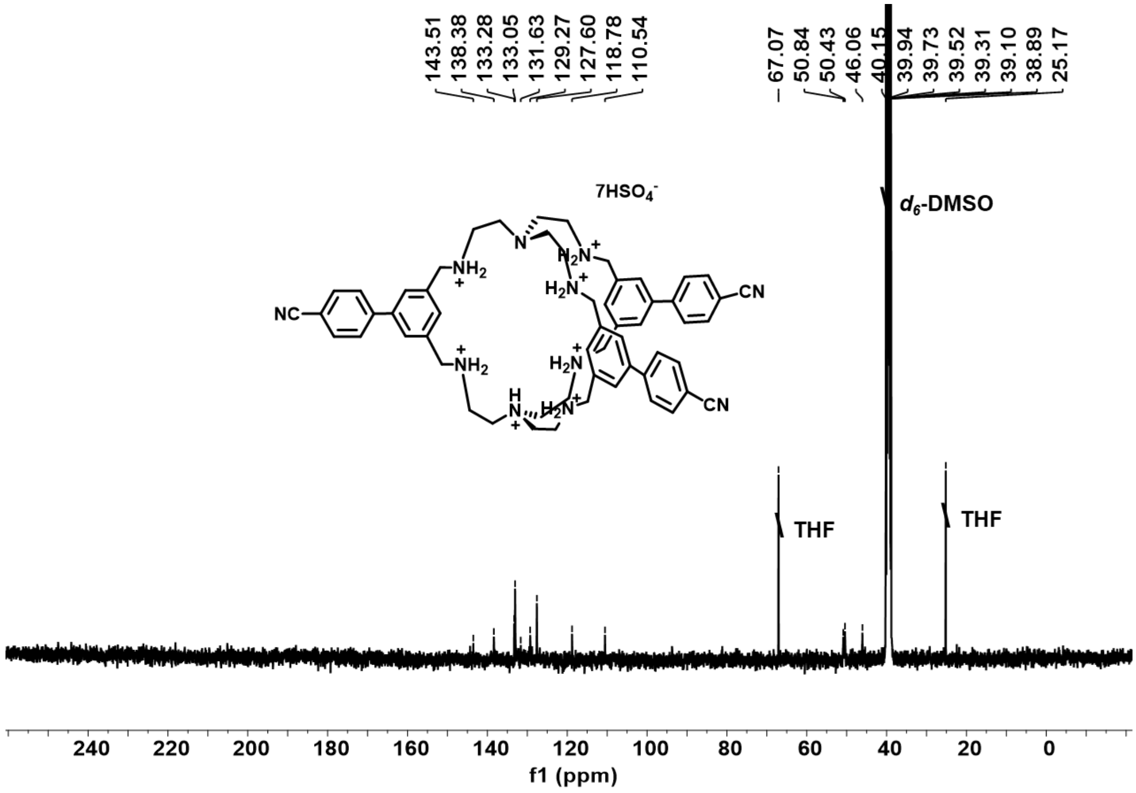

Figure S6. ^{13}C NMR spectrum of Cage1-H_2SO_4 in solution (100 MHz, DMSO-d_6, 25 °C).

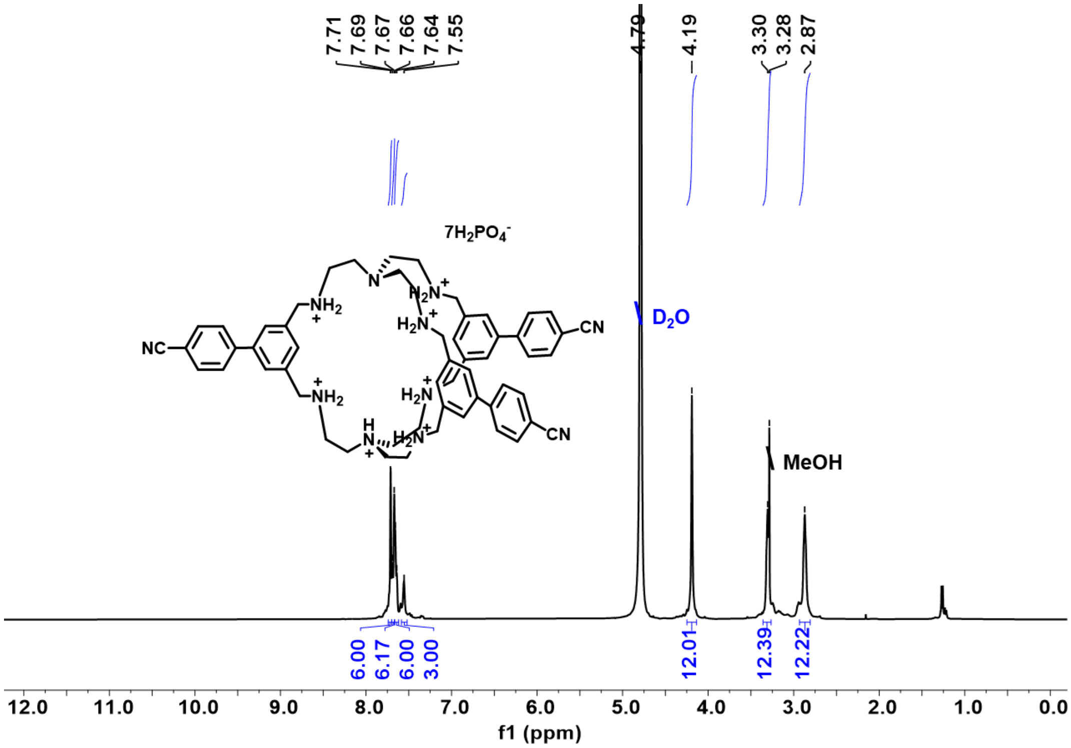

Figure S7. ^{1}H NMR spectrum of Cage1-H_3PO_4 in solution (400 MHz, D_2O, 25 °C).

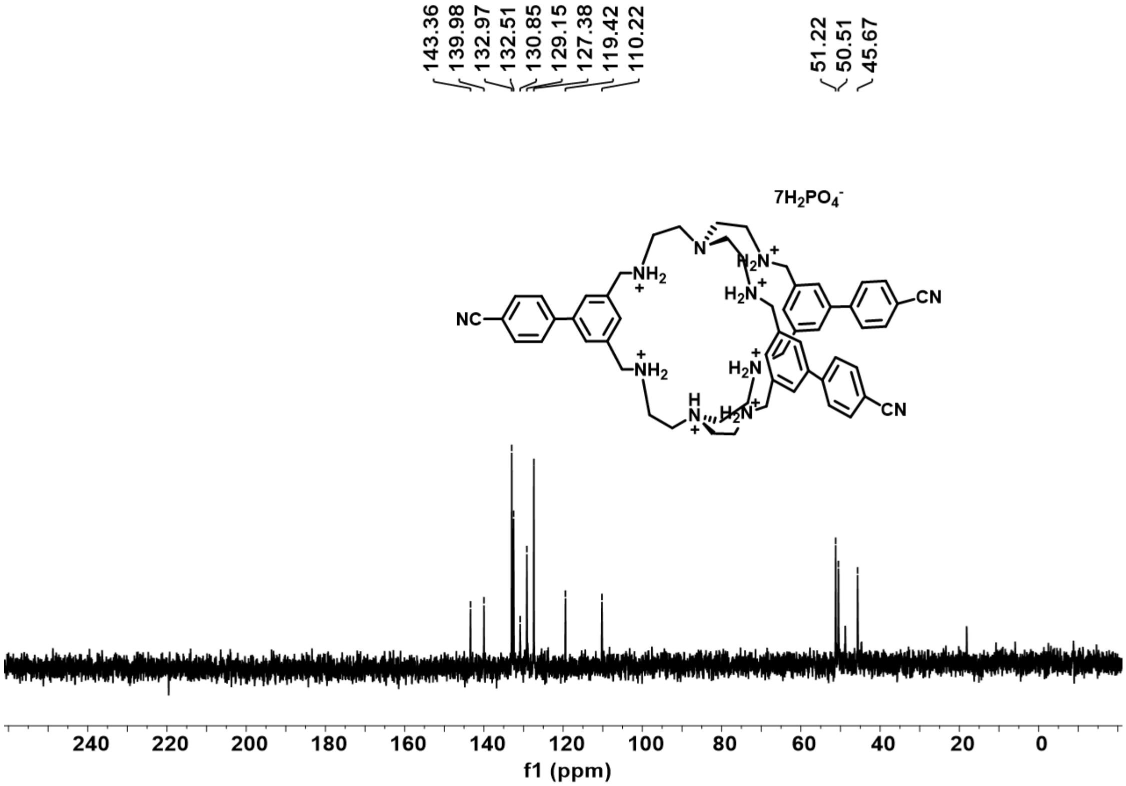

Figure S8. ^{13}C NMR spectrum of Cage1-H_3PO_4 in solution (100 MHz, D_2O, 25 °C).

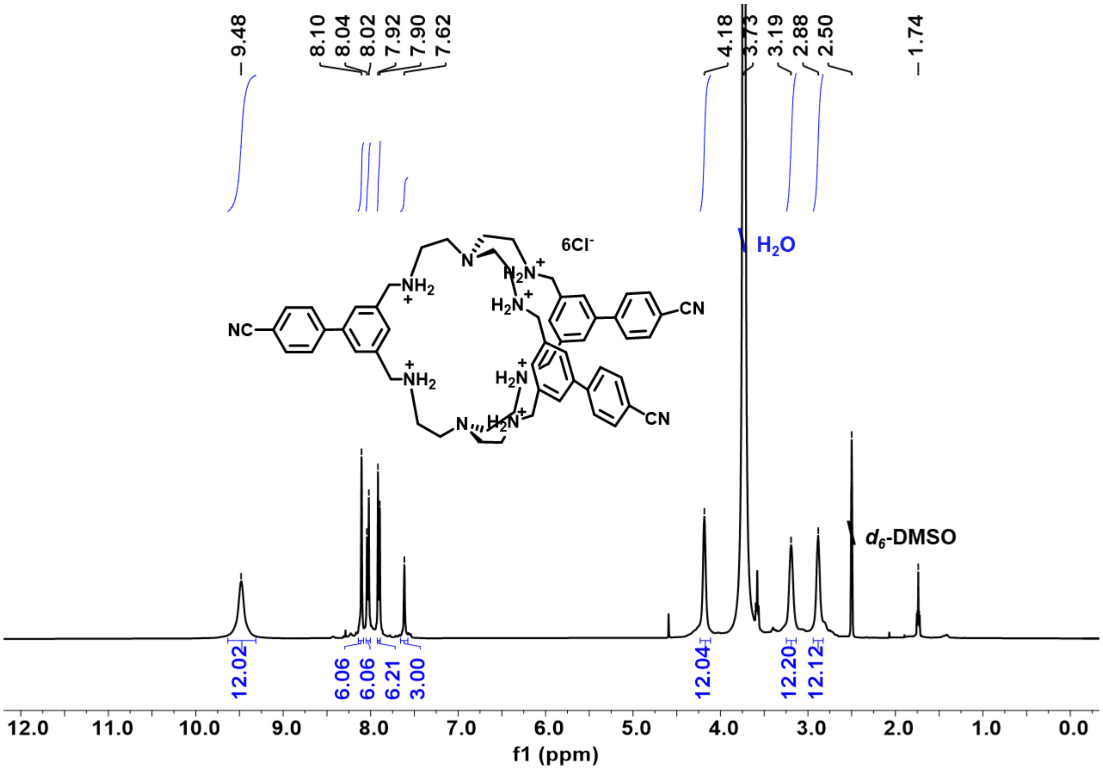

Figure S9. ^{1}H NMR spectrum of Cage1-HCl in solution (400 MHz, DMSO-d_6, 25 °C).

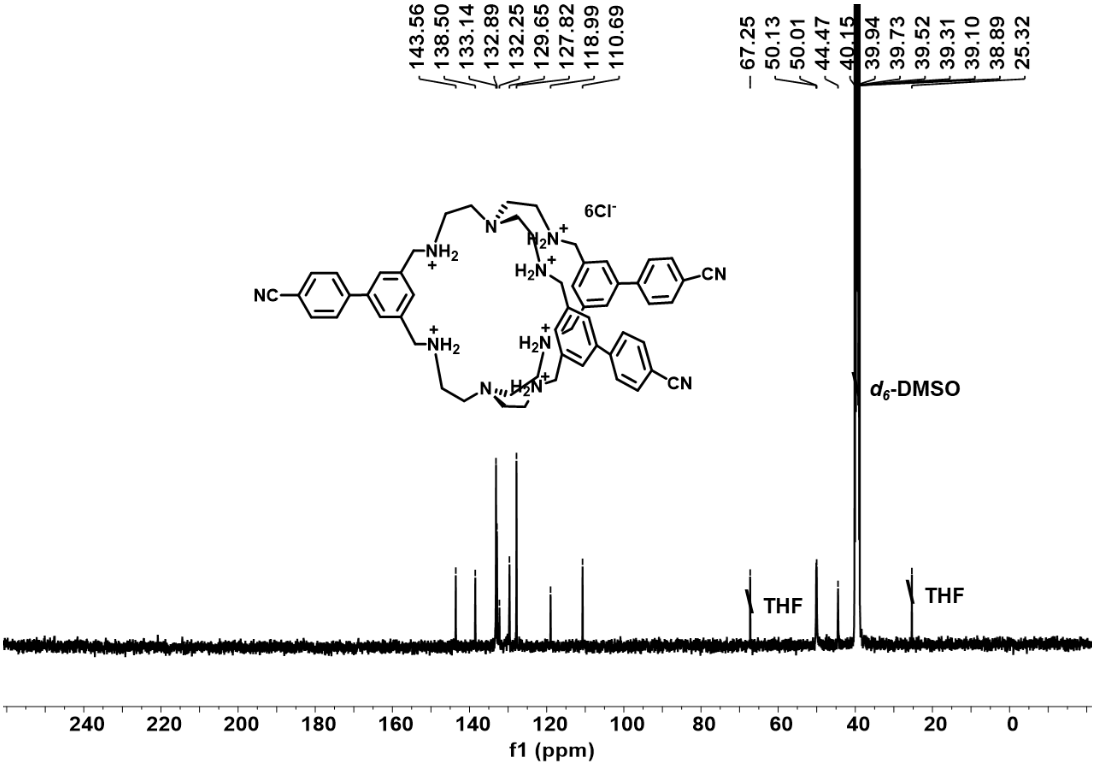

Figure S10. ^{13}C NMR spectrum of Cage1-HCl in solution (100 MHz, DMSO-d_6, 25 °C).

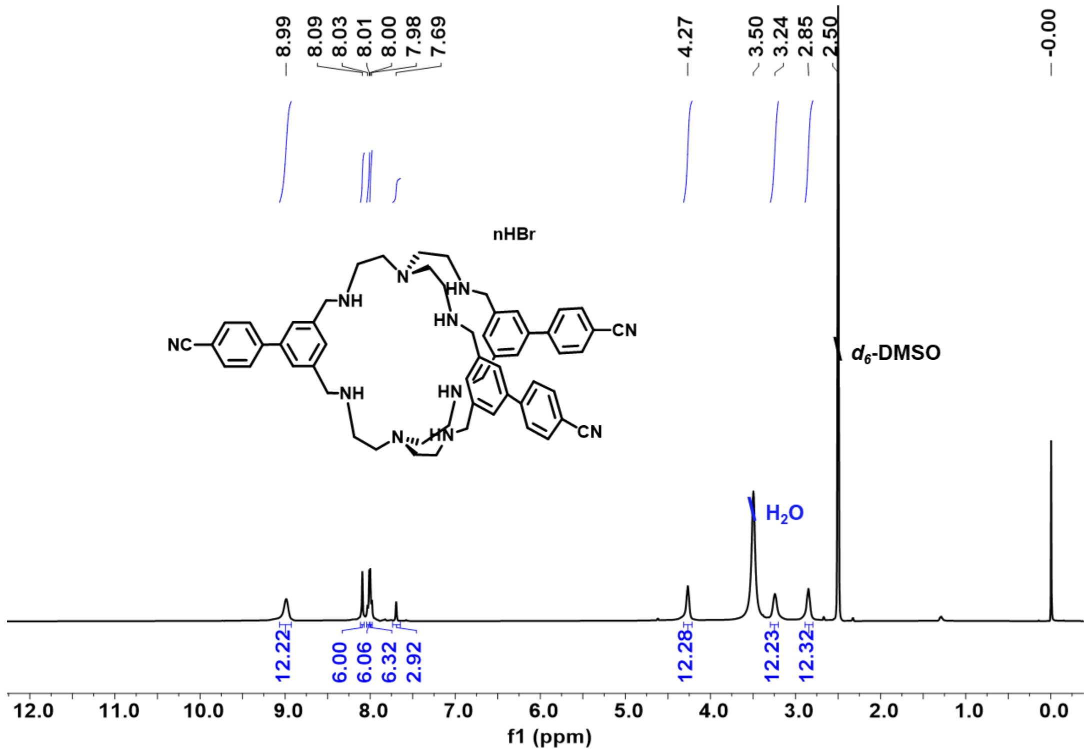

Figure S11. ^{1}H NMR spectrum of Cage1-HBr in solution (400 MHz, DMSO-d_6, 25 °C).

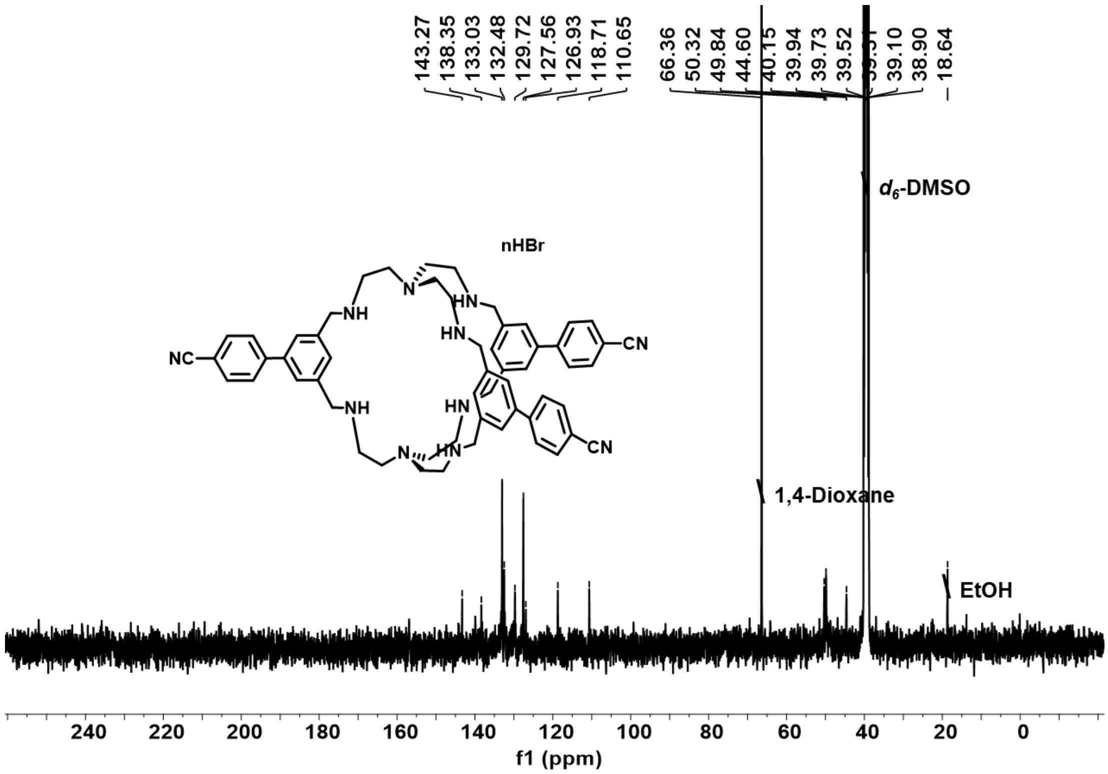

Figure S12. ^{13}C NMR spectrum of Cage1-HBr in solution (100 MHz, DMSO-d_6, 25 °C).

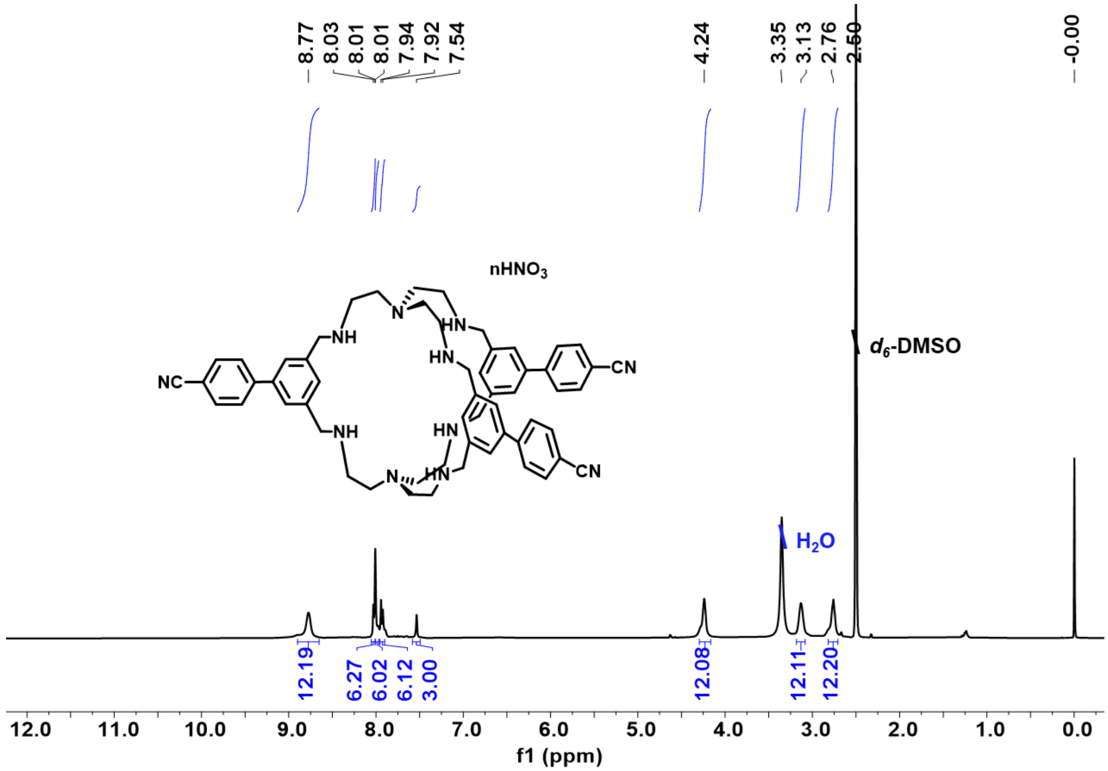

Figure S13. ^{1}H NMR spectrum of Cage1-HNO_3 in solution (400 MHz, DMSO-d_6, 25 °C).

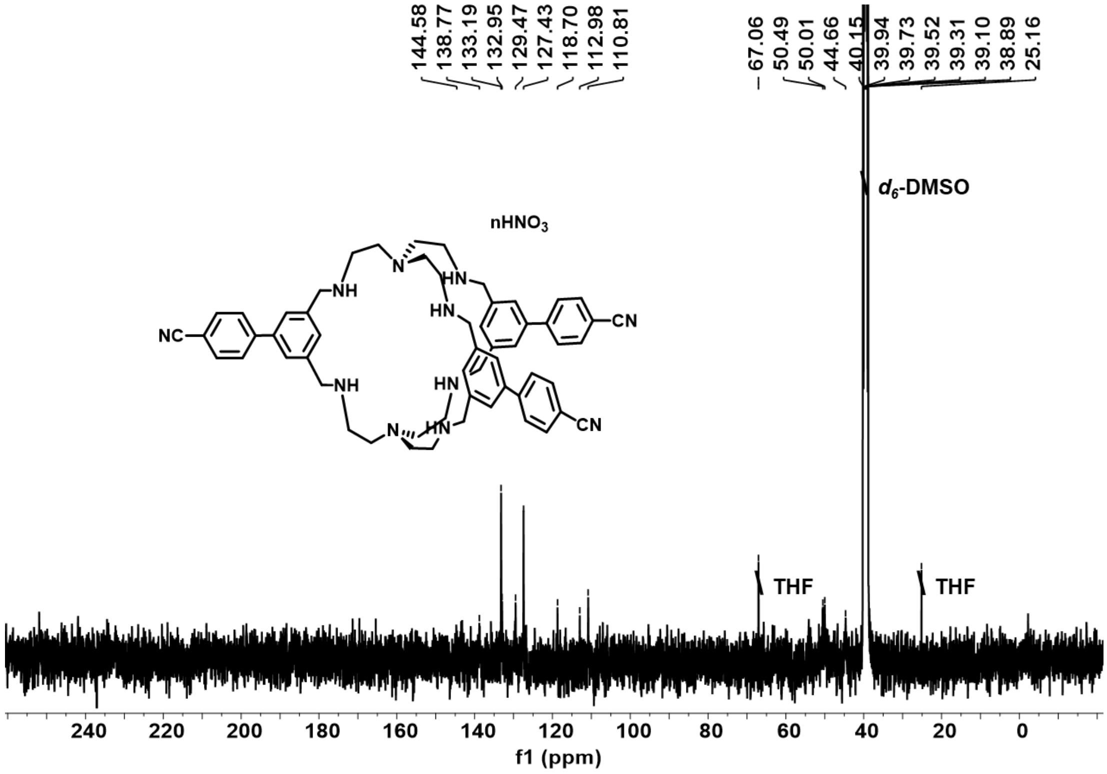

Figure S14. ^{13}C NMR spectrum of Cage1-HNO_3 in solution (100 MHz, DMSO-d_6, 25 °C).

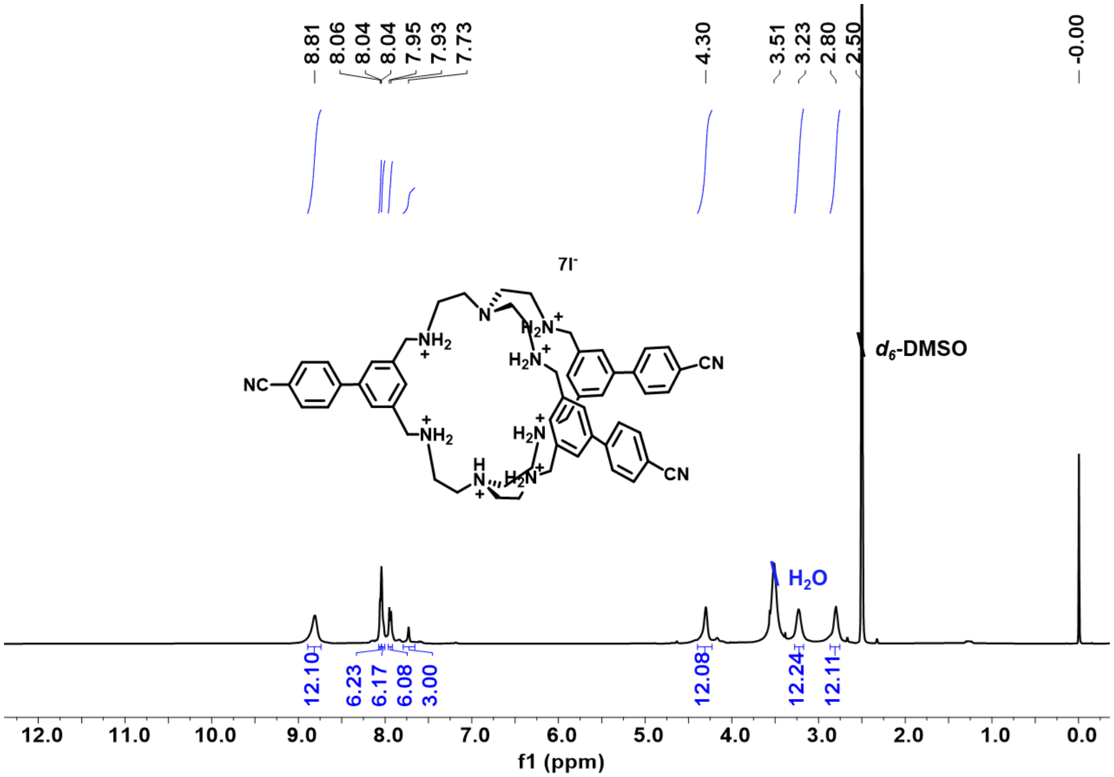

Figure S15. ^{1}H NMR spectrum of Cage1-HI in solution (400 MHz, DMSO-d_6, 25 °C).

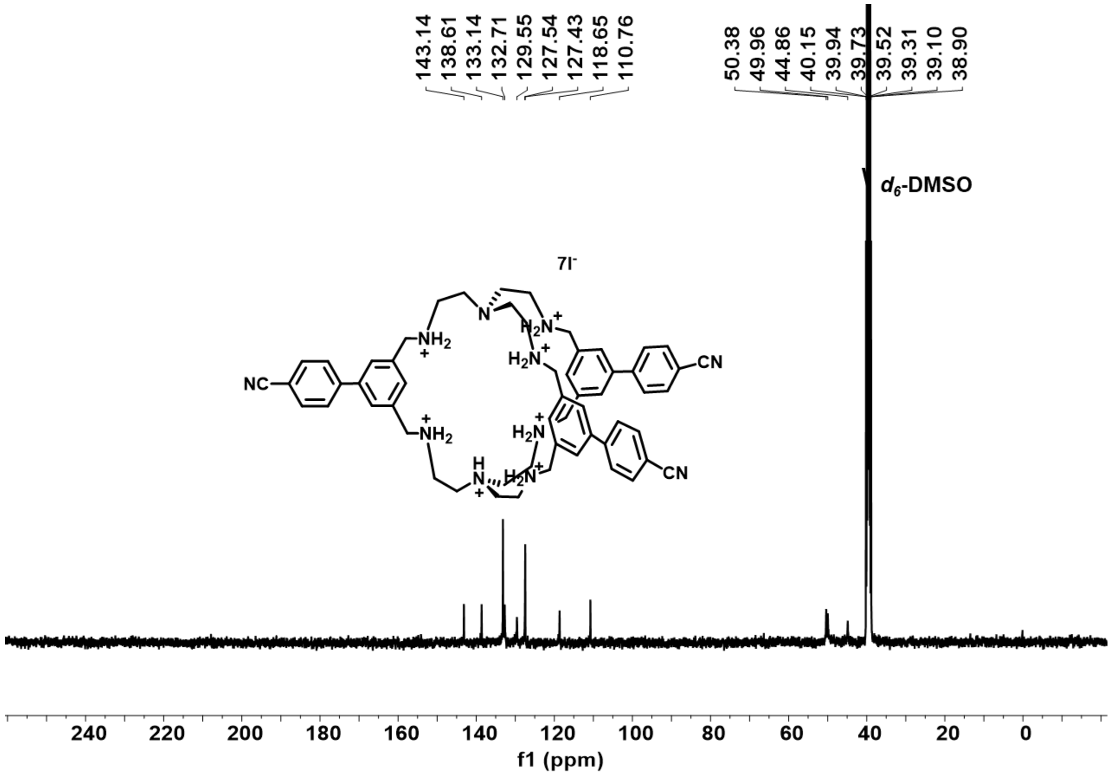

Figure S16. ^{13}C NMR spectrum of Cage1-HI in solution (100 MHz, DMSO-d_6, 25 °C).

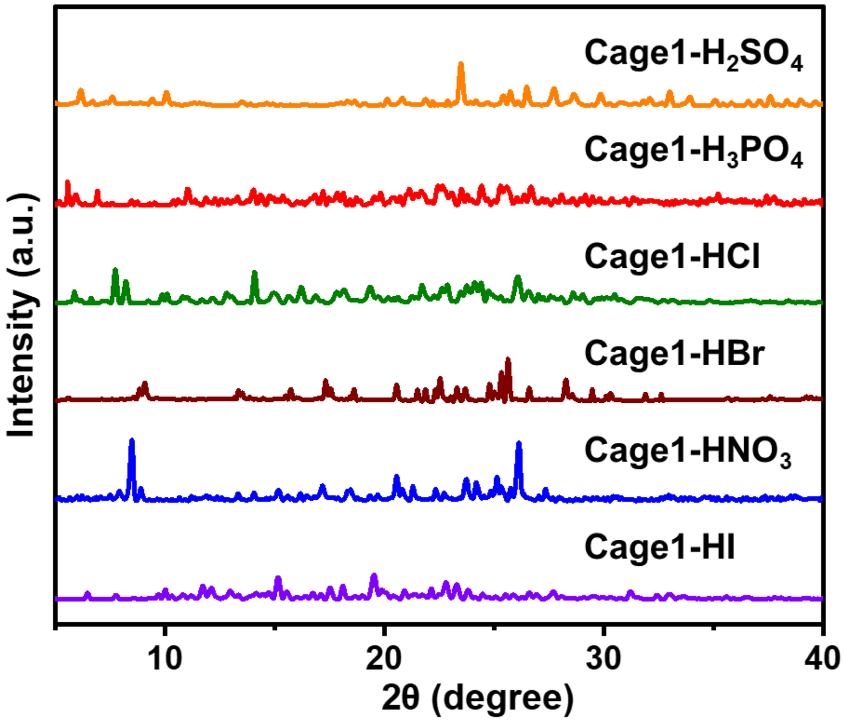

Figure S17. PXRD patterns of Cage1-HX series salts.

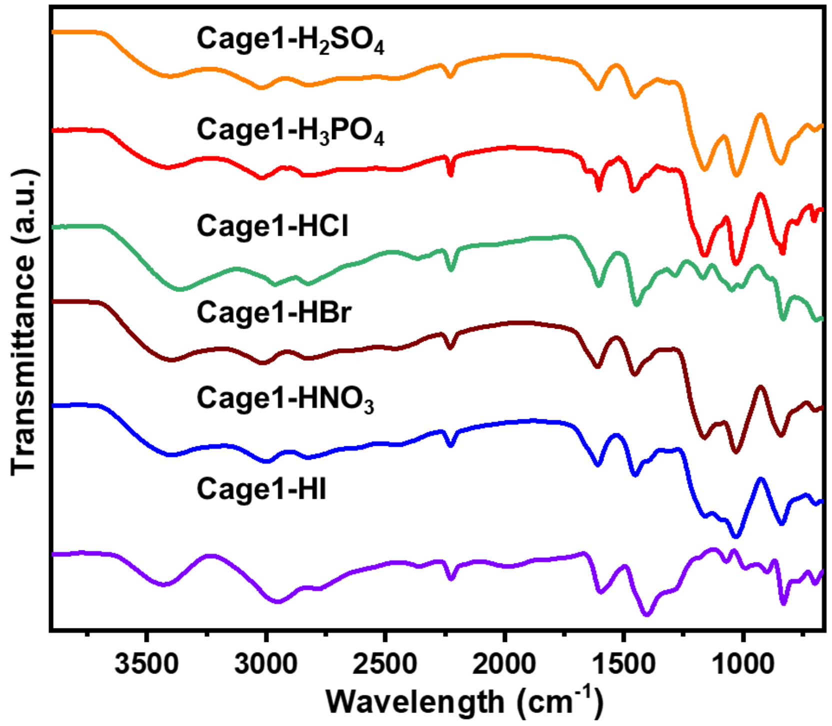

Figure S18. FTIR spectra of Cage1-HX series salts.

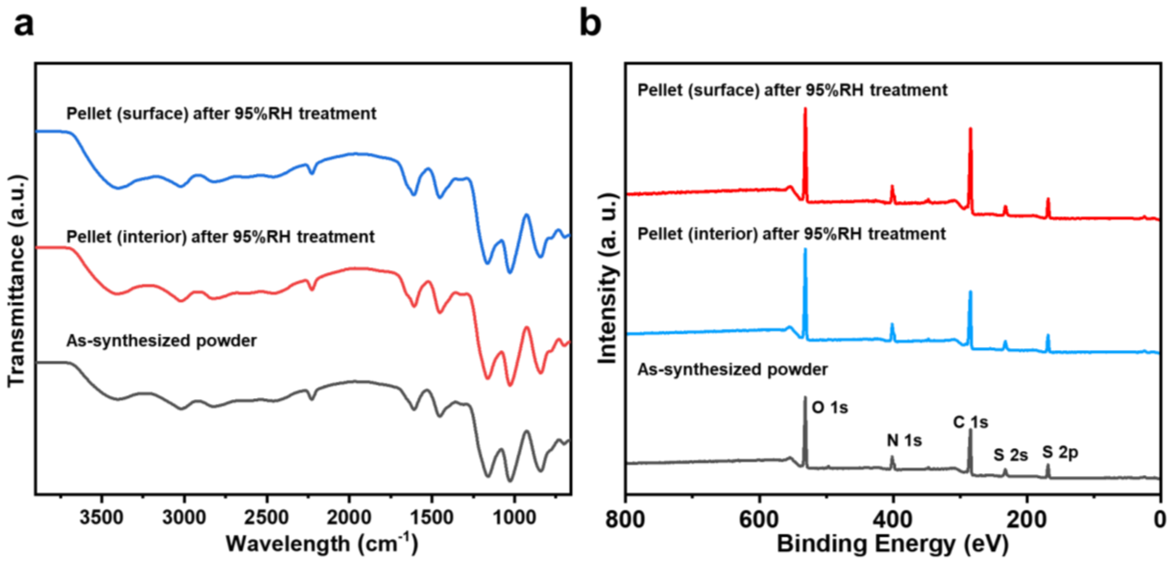

Figure S19. (a) FTIR spectra and (b) XPS spectra of Cage1-H_2SO_4 comparing the as-synthesized powder and the pellet (surface and interior) after 95% RH treatment.

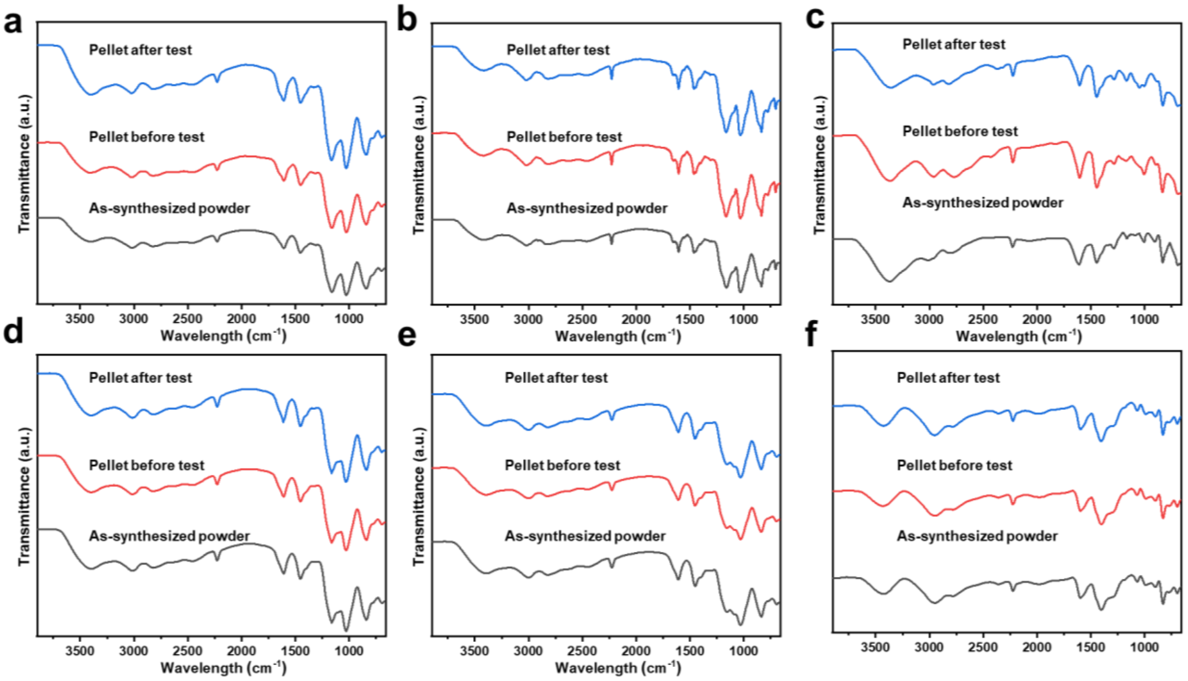

Figure S20. FTIR spectra of (a) Cage1-H_2SO_4, (b) Cage1-H_3PO_4, (c) Cage1-HCl, (d) Cage1-HBr, (e) Cage1-HNO_3, and (f) Cage1-HI comparing the as-synthesized powder and the pellet before and after proton conductivity test.

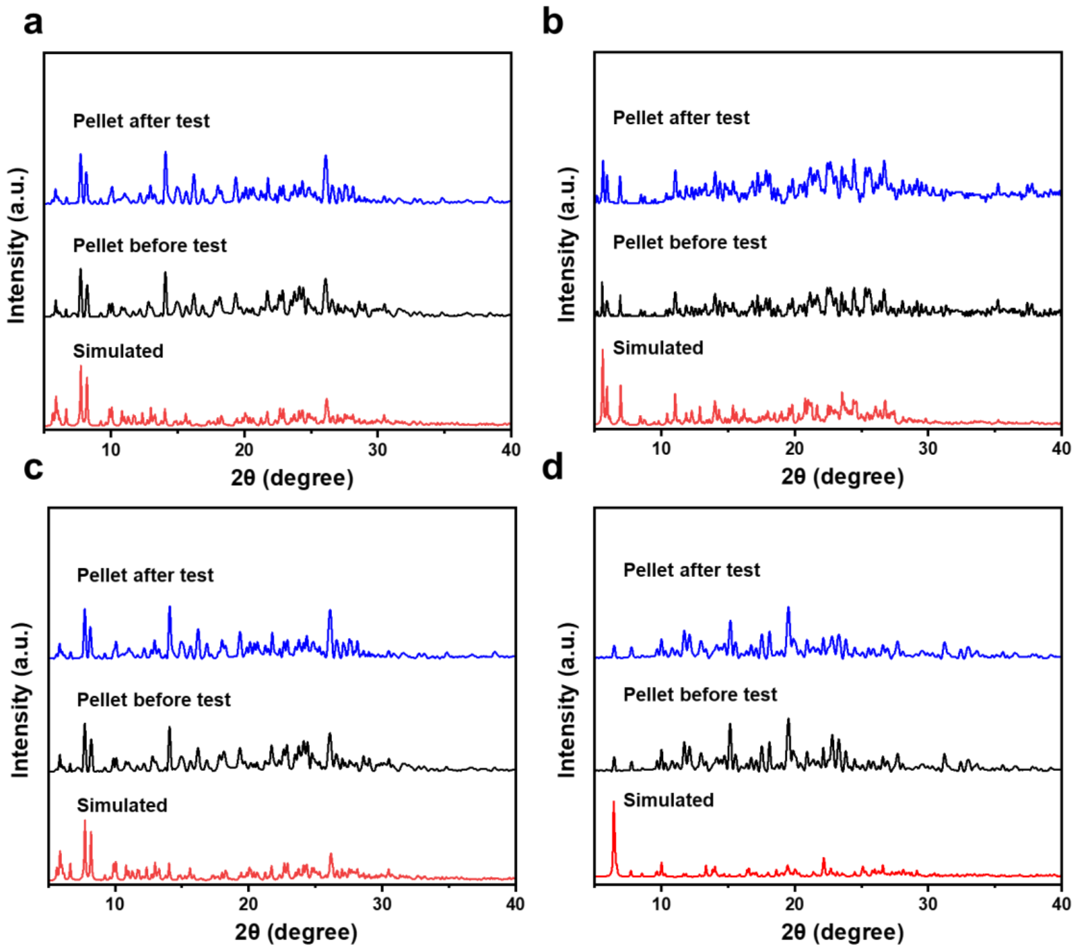

Figure S21. PXRD patterns of the pellet before and after the proton conductivity test, together with the simulated patterns of (a) Cage1-H_2SO_4, (b) Cage1-H_3PO_4, (c) Cage1-HCl, (d) Cage1-HI.

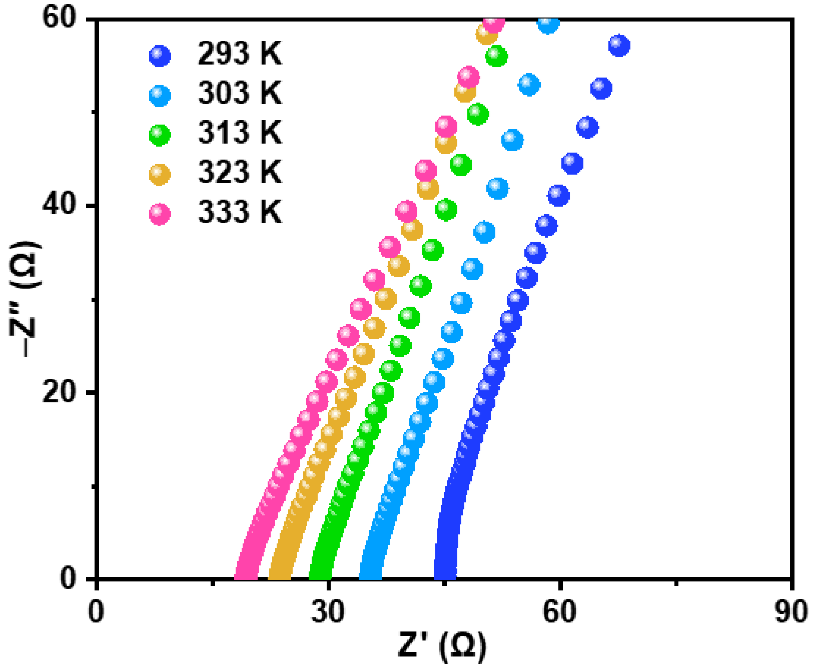

Figure S22. Nyquist plots of Cage1-HBr as a function of temperature under 95% RH.

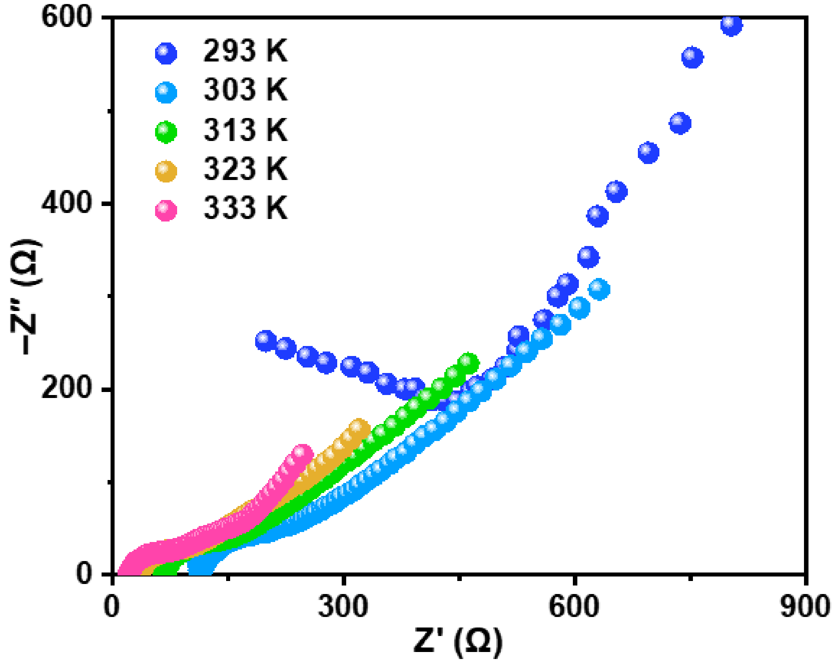

Figure S23. Nyquist plots of Cage1-HNO_3 as a function of temperature under 95% RH.

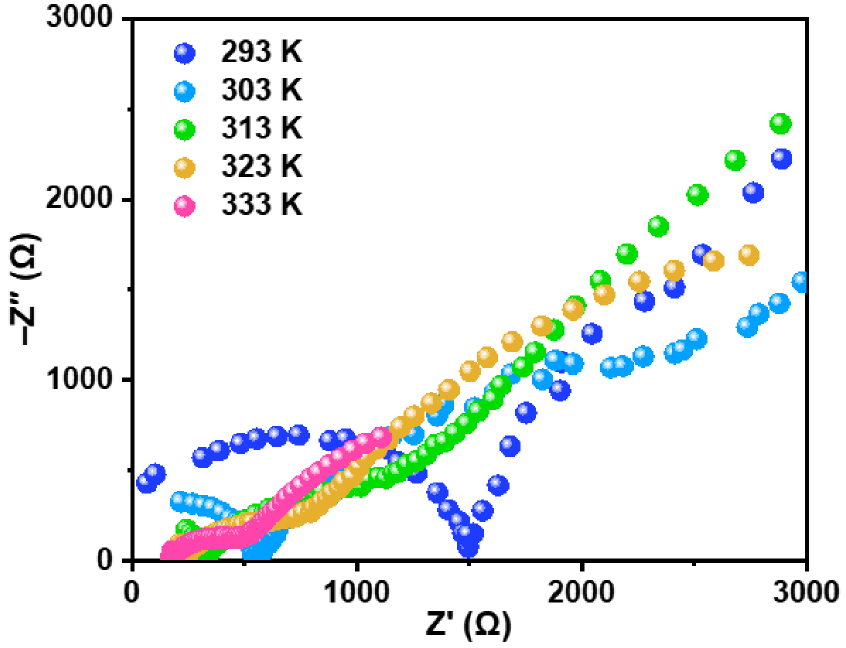

Figure S24. Nyquist plots of Cage1-HI as a function of temperature under 95% RH.

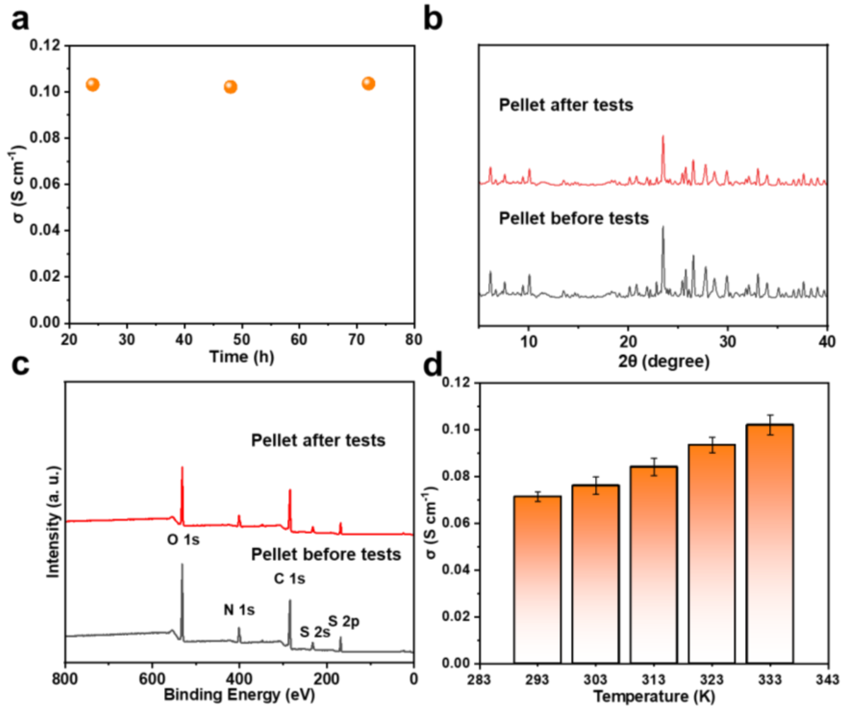

Figure S25. (a) Long-term proton conductivity tests of Cage1-H_2SO_4 under 95% RH at 333 K, and its (b) PXRD patterns and (c) XPS spectra of the pellet before and after the proton conductivity tests. (d) Temperature-dependent proton conductivity of Cage1-H_2SO_4 under 95% RH demonstrating reproducibility. Error bars represent standard deviation from three independent measurements.

Table S1. Proton conductivity of Cage1 series at different temperature under 95% RH.

Proton conductivity ($S\ cm^{-1}$)	293 K	303 K	313 K	323 K	333 K
**Cage1-H_2SO_4**	7.31×10^{-2}	7.93×10^{-2}	8.73×10^{-2}	9.66×10^{-1}	1.03×10^{-1}
**Cage1-H_3PO_4**	1.45×10^{-2}	1.87×10^{-2}	2.02×10^{-2}	2.50×10^{-2}	2.83×10^{-2}
**Cage1-HCl**	3.09×10^{-3}	3.40×10^{-3}	4.24×10^{-3}	5.36×10^{-3}	5.99×10^{-3}
**Cage1-HBr**	2.26×10^{-3}	2.87×10^{-3}	3.51×10^{-3}	4.30×10^{-3}	5.29×10^{-3}
**Cage1-HNO_3**	1.91×10^{-4}	4.77×10^{-4}	6.37×10^{-4}	8.49×10^{-4}	9.55×10^{-4}
**Cage1-HI**	5.57×10^{-5}	1.48×10^{-4}	2.78×10^{-4}	3.88×10^{-4}	4.95×10^{-4}

Table S2. Proton conductivity of Cage1 series under different RH at 293 K.

Proton conductivity ($S\ cm^{-1}$)	67% RH	75% RH	85% RH	95% RH
**Cage1-H_2SO_4**	2.48×10^{-3}	2.30×10^{-2}	4.07×10^{-2}	7.31×10^{-2}
**Cage1-H_3PO_4**	3.40×10^{-4}	7.28×10^{-4}	1.23×10^{-3}	1.45×10^{-2}
**Cage1-HCl**	1.82×10^{-4}	3.64×10^{-4}	8.15×10^{-4}	3.09×10^{-3}
**Cage1-HBr**	1.27×10^{-6}	1.70×10^{-4}	6.65×10^{-4}	2.26×10^{-3}
**Cage1-HNO_3**	6.47×10^{-7}	1.91×10^{-6}	4.77×10^{-6}	1.91×10^{-4}
**Cage1-HI**	6.37×10^{-7}	1.11×10^{-6}	1.49×10^{-6}	5.57×10^{-5}

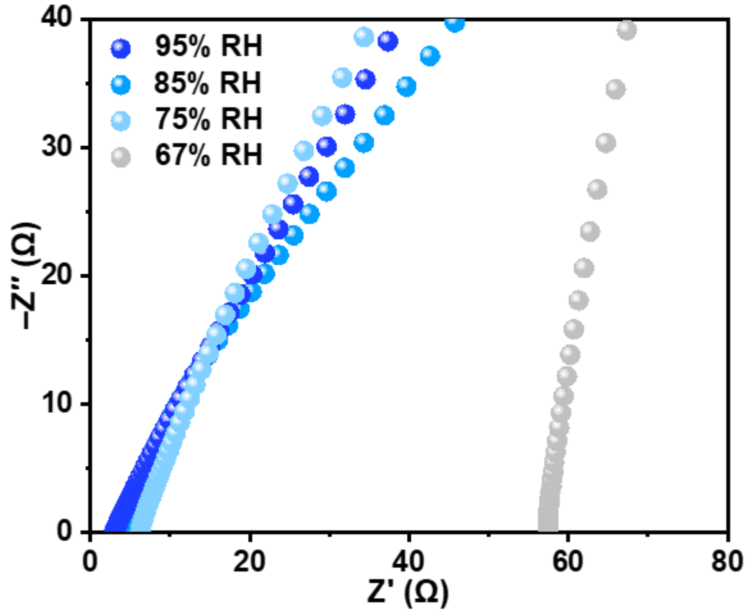

Figure S26. Nyquist plots of Cage1-H_2SO_4 under different RH at 293 K.

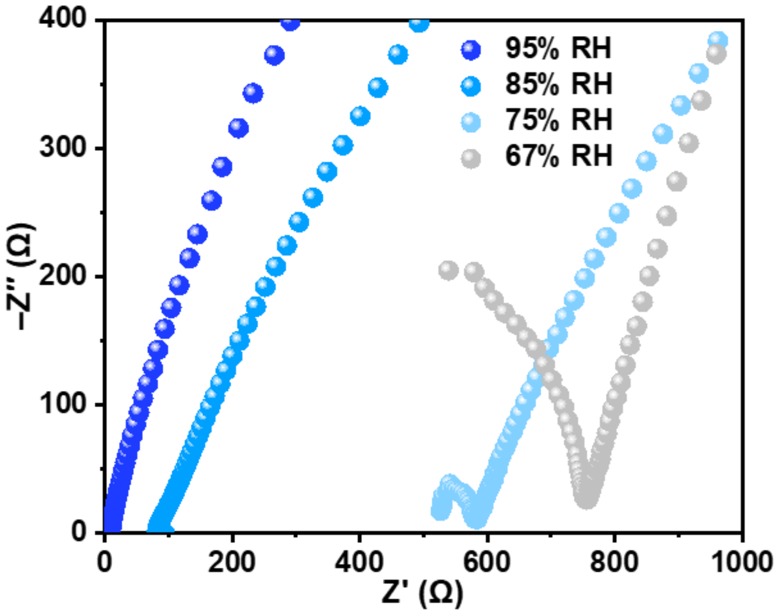

Figure S27. Nyquist plots of Cage1-H_3PO_4 under different RH at 293 K.

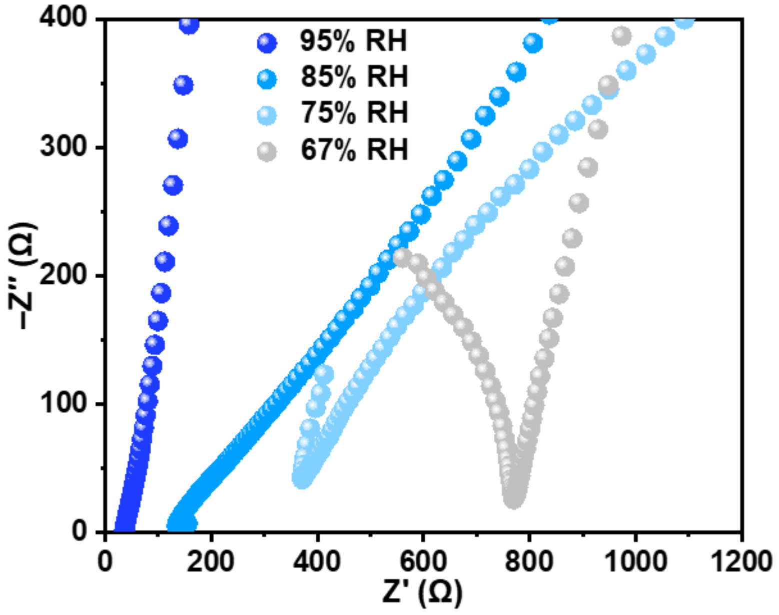

Figure S28. Nyquist plots of Cage1-HCl under different RH at 293 K.

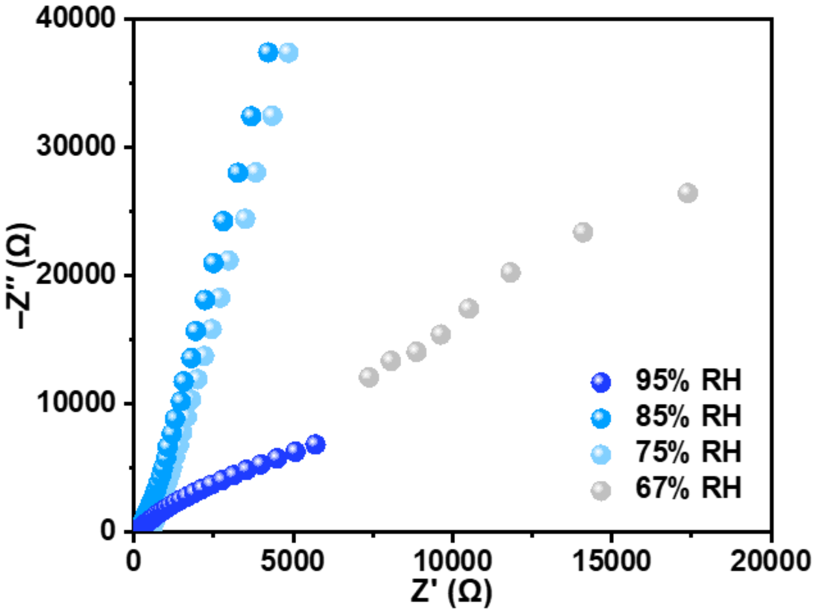

Figure S29. Nyquist plots of Cage1-HBr under different RH at 293 K.

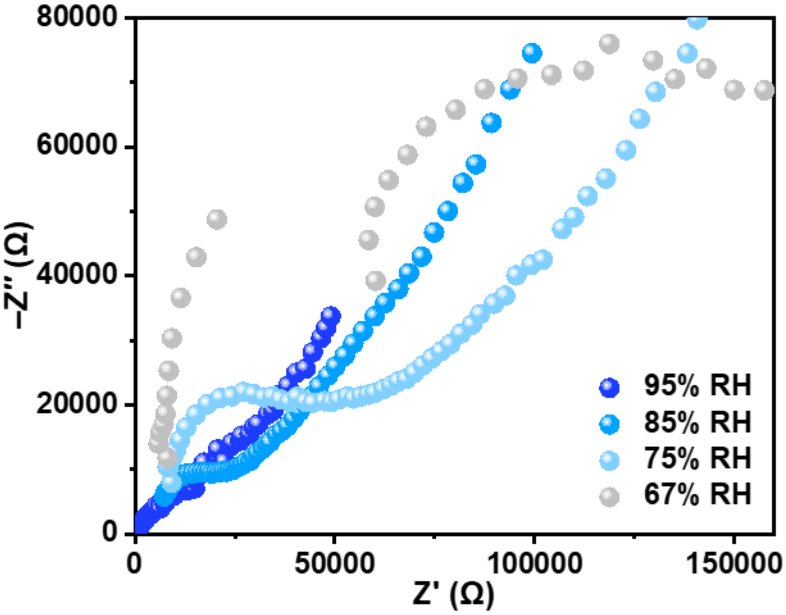

Figure S30. Nyquist plots of Cage1-HNO_3 under different RH at 293 K.

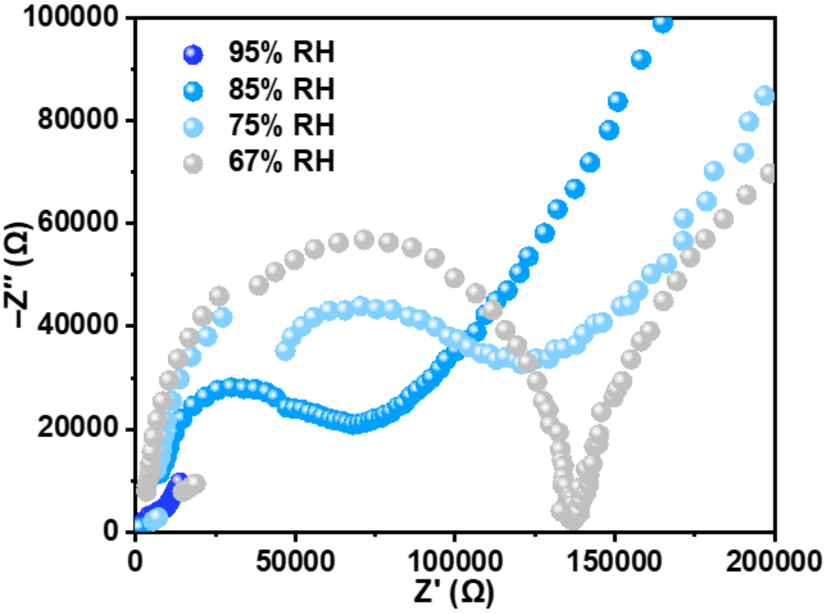

Figure S31. Nyquist plots of Cage1-HI under different RH at 293 K.

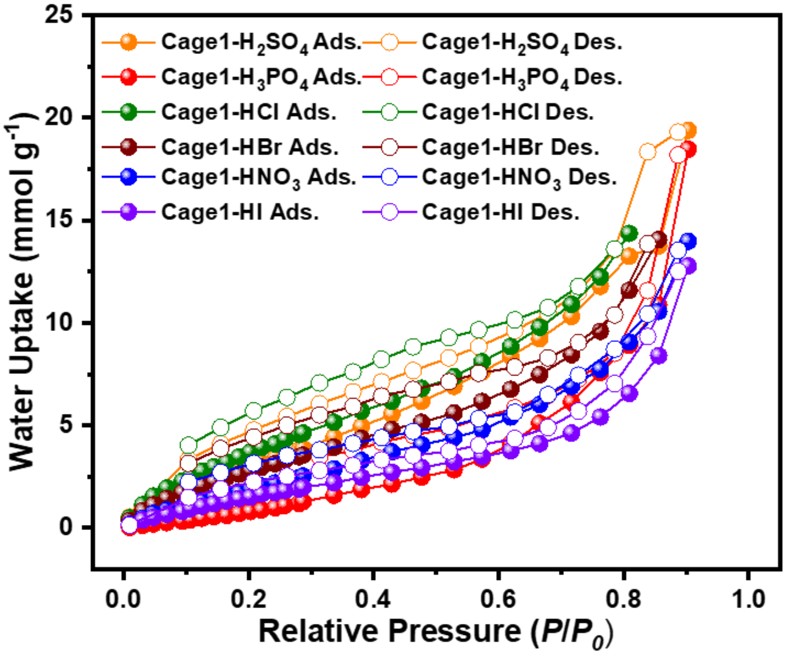

Figure S32. Water vapor adsorption/desorption isotherms of Cage1 series.

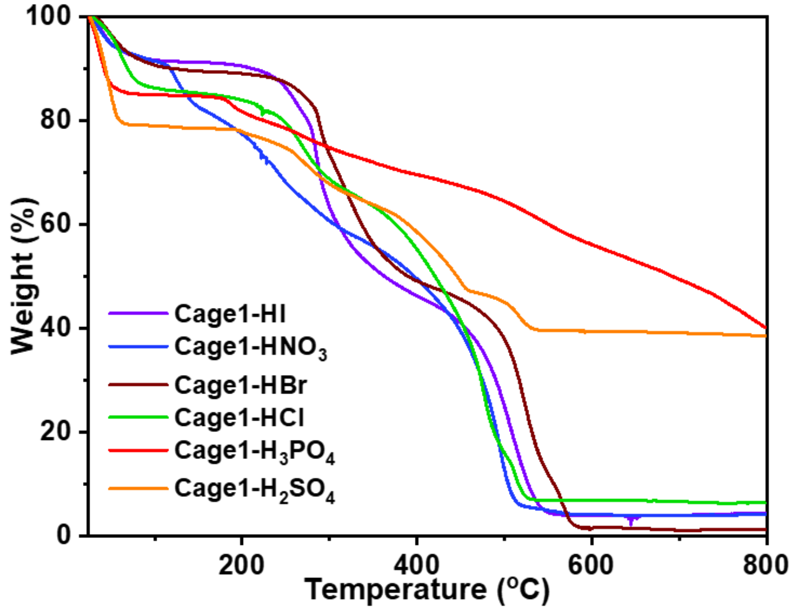

Figure S33. TGA profiles of Cage1 series powders after adsorption under 95% RH.

Table S3. Proton conductivity vs *Ea*, and test conditions of various supramolecular hosts reported in the literature.

Material	Proton conductivity (S cm^{-1})	*Ea* (eV)	Conditions	Reference
$CB[6]\cdot 1.1HCl\cdot 11.3H_2O$	1.1×10^{-3}	0.39	298 K, 98% RH	2
$CB[6]\cdot 1.2H_2SO_4\cdot 6.4H_2O$	1.3×10^{-3}	0.31	298 K, 98% RH	
$CB[8]\cdot 6.8HCO_2H\cdot 13H_2O$	1.3×10^{-4}	0.56	298 K, 98% RH	
$(H_{12}RCC1)^{12+}\cdot 6(Cl)^{2-}\cdot 4H_2O$	1.1×10^{-3}	0.35	303 K, 95% RH	3
$(H_{12}RCC1)^{12+}\cdot 6(SO_4)^{2-}\cdot 27.25H_2O$	6.1×10^{-5}	0.10	303 K, 95% RH	
Organic cages diphanes-TFA	1.37×10^{-5}	0.39	333 K, 48% RH	4
TFA@amine cage 1	1.6×10^{-3}	0.16	303 K, 48% RH	5
TFA@amine cage 2	5.23×10^{-5}	0.45	303 K, 48% RH	
Cage-1	7.67×10^{-4}	0.032	343 K, 85% RH	6
Cage-2	2.68×10^{-4}	0.50	343 K, 85% RH	
TA	4.61×10^{-5}	0.97	343 K, 85% RH	
Cage1-H_2SO_4	1.03×10^{-1}	0.075	333 K, 95% RH	This work
Cage1-H_3PO_4	2.83×10^{-2}	0.14	333 K, 95% RH	
Cage1-HCl	5.99×10^{-3}	0.15	333 K, 95% RH	
Cage1-HBr	5.29×10^{-3}	0.17	333 K, 95% RH	
Cage1-HNO_3	9.55×10^{-4}	0.33	333 K, 95% RH	
Cage1-HI	4.95×10^{-4}	0.46	333 K, 95% RH	

Table S4. Proton conductivity vs *Ea*, and test conditions of state-of-the-art materials reported in the literature.

Material	Proton conductivity (S cm^{-1})	*Ea* (eV)	Conditions	Reference
p-6PA-HPB	2.5×10^{-2}		298 K, 95% RH	7
CB[6]·1.1HCl·11.3H_2O	1.1×10^{-3}	0.39	298 K, 98% RH	2
CB[6]·1.2H_2SO_4·6.4H_2O	1.3×10^{-3}	0.31		
CB[8]·6.8HCO_2H·13H_2O	1.3×10^{-4}	0.56		
UiO-66(SO3H)2	8.4×10^{-2}	0.32	353 K, 90% RH	8
Sulfonated polymer 1S	7.72×10^{-2}	0.34	353 K, 90% RH	9
HOF-GS-11	1.8×10^{-2}	0.135	303 K, 95% RH	10
$(H_{12}RCC1)^{12+}$·6$(Cl)^{2-}$·4H_2O	1.1×10^{-3}	0.25	303 K, 95% RH	3
$(H_{12}RCC1)^{12+}$·6$(SO_4)^{2-}$·27.25H_2O	6.1×10^{-5}	0.10		
(Me2NH2)[Eu(L)]	1.25×10^{-3}	0.21	373 K, 98% RH	11
PTSA@TpAzo	7.8×10^{-2}	0.11	353 K, 95% RH	12
CPOS-2	2.2×10^{-2}	0.61	333 K, 98% RH	13
MIP-202(Zr)	1.1×10^{-2}	0.22	363 K, 95% RH	14
BIP	3.2×10^{-2}	0.31	368 K, 95% RH	15
SSP@ZIF-8 membrane	5×10^{-2}	1.09	348 K, 95% RH	16
$CdPS_3$ nanosheets-based membrane	9.5×10^{-1}	0.177	363 K, 98% RH	17
H_3PO_4@COF	1.91×10^{-1}	0.34	433 K, anhydrous	18
H_3PO_4@Perfluoroalkyl COF, COF-F6-H	2.64×10^{-2}	0.12	413 K, anhydrous	19
MOF-74(Mg)−Urea	3.7×10^{-2}	0.12	323 K, 95% RH	19
Organice cage diphane, endo-[1,2,5]diphane–TFA	1.37×10^{-5}	0.39	333 K, 48% RH	4
PA@Pz-FAN	1.16×10^{-2}	0.18	353 K, 93% RH	20
TFPPY-BT-COF-H2PO3	1.12×10^{-3}	0.20	333 K, 98% RH	21
[Pt2(MPC)4Cl2Co-(DMA)(HDMA)·guest]	2.2×10^{-2}	0.33	333 K, 95% RH	22

TFA@amine cage 1	1.6×10^{-3}	0.16	303 K, 98% RH	5
TFA@amine cage 2	5.23×10^{-5}	0.45		
ZrTSaT	3.7×10^{-1}	1.18	363 K, 90% RH	23
iHOF-9	4.38×10^{-2}	0.16	363 K, 98% RH	24
HOF-FJU-36	1.38×10^{-4}	0.66	298 K, 80% RH	25
Zn-HHTP-H2O	1.6×10^{-5}	0.14	343 K, 95% RH	26
H3PO4@CTF-TF	1.82×10^{-1}	0.37	423 K, anhydrous	27
H3PO4@NKCOF-54	2.33×10^{-2}	0.29	433 K, anhydrous	28
8HSA@MIL-101	3.06×10^{-1}	0.22	358 K, 98% RH	29
Ti-dobdc–LiI	1.88×10^{-2}	0.15	298 K, 90% RH	30
gigantic {Mo132}	9.01×10^{-2}	0.38	353 K, 98% RH	31
Mn-HOF-FA	2×10^{-2}	0.41	333 K, 75% RH	32
$PIL_{0.5}$@mTpPa-SO_3H	1.02×10^{-1}	0.24	363 K, 100% RH	33
Cage-1	7.67×10^{-4}	0.032	343 K, 95% RH	6
Cage-2	2.68×10^{-4}	0.50	343 K, 85% RH	
TA	4.61×10^{-5}	0.97	343 K, 85% RH	
TPMA-3F/MTBPS	1.34×10^{-2}	1.01	363 K, 95% RH	34
TTBT.Br	1.1×10^{-1}	0.33	343 K, 90% RH	35
TTBT.Cl	2.9×10^{-2}	0.69	343 K, 90% RH	
Cage1-H_2SO_4	1.03×10^{-1}	0.075	333 K, 95% RH	This work

Single Crystal X-ray Diffraction and Analysis

The crystals of Cage1-H_2SO_4, Cage1-H_3PO_4, Cage1-HCl and Cage1-HI suitable for single crystal diffraction were obtained from water/MeOH/DCM by slow evaporation at room temperature after several days.

Single crystal X-ray diffraction (SCXRD) data were recorded on a Bruker D8-Venture single crystal X-ray diffractometer equipped with a digital camera diffractometer using graphite-monochromated Ga-Kα or Mo-Kα radiation at 120 K. Data integration and reduction were performed using the SaintPlus 6.01 software. The absorption corrections and correction of other systematic errors were performed by the multi-scan method implemented in SADABS.[36] Structures were solved using Direct Methods (SHELXS-97) and refined using the SHELXL-2014[37] program package (full-matrix least squares on F^2) contained in OLEX2 and X-Seed.[38-39] In all cases the non-hydrogen atoms were refined anisotropically. The hydrogen atoms were fixed geometrically using riding atom model. The crystal data and refinement conditions for Cage1-H_2SO_4, Cage1-H_3PO_4, Cage1-HCl and Cage1-HI crystals are reported in Tables S5-S8. Several alerts arise from weak diffraction, structural disorder, the large asymmetric unit, and overall data quality. Each alert has been carefully examined, and reasonable explanations are provided in the checkCIF report. Further details may be obtained from the cif, which may be obtained from the Cambridge Crystallographic Data Centre (CCDC) upon reference to CCDC numbers 2527525 (Cage1-H_2SO_4), 2425249 (Cage1-H_3PO_4), 2425250 (Cage1-HCl), and 2527526 (Cage1-HI).

Table S5. Crystallographic details for Cage1-H_2SO_4.

IDENTIFICATION CODE	CageCNH$_2$SO$_4$
**Empirical formula**	$C_{59}H_{95.80}N_{11}O_{35.61}S_7$
**Formula weight (g/mol)**	1753.52
**Temperature /K**	120
**Crystal system**	monoclinic
**Space group**	P 1 21/n 1
***a*/ Å**	15.0335(9)
***b*/ Å**	15.4989(8)
***c*/ Å**	35.684(2)
**α/°**	90
**β/°**	98.044(2)
**γ/°**	90
**Volume/ Å^3**	8232.6(8)
**Z**	4
**ρcalcg/cm^3**	1.415
**F(000)**	3695.0
**Radiation**	MoKα (λ = 0.71073)
**reflections collected**	20431
**Independent reflections**	9945 [R_{int} = 0.1595]
**Data/restraints/para meters**	8599/381/958
**Goodness-of-fit on F2**	1.206
**Final R indexes [I>=2σ (I)]**	$R1$ = 0.1653, $wR2$ = 0.5303
**CCDC numbers**	2527525

Table S6. Crystallographic details for Cage1-H_3PO_4.

IDENTIFICATION CODE	CageCNH3PO4
**Empirical formula**	$C_{114}H_{188.60}N_{22}O_{63.60}P_{13.10}$
**Formula weight (g/mol)**	3290.76
**Temperature /K**	120(2)
**Crystal system**	triclinic
**Space group**	P-1
***a*/ Å**	15.9808(10)
***b*/ Å**	17.2671(10)
***c*/ Å**	32.493(2)
**α/°**	100.124(3)
**β/°**	95.293(3)
**γ/°**	108.932(3)
**Volume/ Å^3**	8241.2(9)
**Z**	2
**ρcalcg/cm^3**	1.326
**F(000)**	3464.0
**Radiation**	GaKα (λ = 1.34139)
**reflections collected**	28789
**Independent reflections**	9858 [R_{int} = 0.0868]
**Data/restraints/para meters**	21021/1211/1989
**Goodness-of-fit on F2**	1.255
**Final R indexes [I>=2σ (I)]**	*R*1 = 0.1361, *wR*2 = 0.4360
**CCDC numbers**	2425249

Table S7. Crystallographic details for Cage1-HCl.

IDENTIFICATION CODE	CageCNHCl
**Empirical formula**	$C_{230}H_{383.2}Cl_{23.4}N_{44}O_{54.6}$
**Formula weight (g/mol)**	5468.11
**Temperature /K**	293(2)
**Crystal system**	monoclinic
**Space group**	Pc
***a*/ Å**	27.419(3)
***b*/ Å**	18.392(2)
***c*/ Å**	30.924(4)
**α/°**	90
**β/°**	103.912(5)
**γ/°**	90
**Volume/ $Å^3$**	15137(3)
**Z**	2
**ρcalcg/cm^3**	1.200
**F(000)**	5812.0
**Radiation**	GaKα (λ = 1.34138)
**reflections collected**	51618
**Independent reflections**	512 [R_{int} = 0.0905]
**Data/restraints/para meters**	36865/2175/3144
**Goodness-of-fit on F2**	1.079
**Final R indexes [I>=2σ (I)]**	R_1 = 0.1291, wR_2 = 0.3860
**CCDC numbers**	2425250

Table S8. Crystallographic details for Cage1-HI.

IDENTIFICATION CODE	CageCNHI
**Empirical formula**	$C_{60}H_{89}I_{7.23}N_{11}O_7$
**Formula weight (g/mol)**	1993.90
**Temperature /K**	120
**Crystal system**	triclinic
**Space group**	P -1
***a*/ Å**	10.7052(5)
***b*/ Å**	14.1919(7)
***c*/ Å**	27.9450(12)
**α/°**	98.614(2)
**β/°**	98.930(2)
**γ/°**	99.816(2)
**Volume/ Å^3**	4064.4(3)
**Z**	2
**ρcalcg/cm^3**	1.629
**F(000)**	1930.0
**Radiation**	MoKα (λ = 0.71073)
**reflections collected**	14276
**Independent reflections**	9204 [R_{int} = 0.1455]
**Data/restraints/para meters**	7726/666/769
**Goodness-of-fit on F2**	1.223
**Final R indexes [I>=2σ (I)]**	R_1 = 0.1201, wR_2 = 0.3724
**CCDC numbers**	2527526

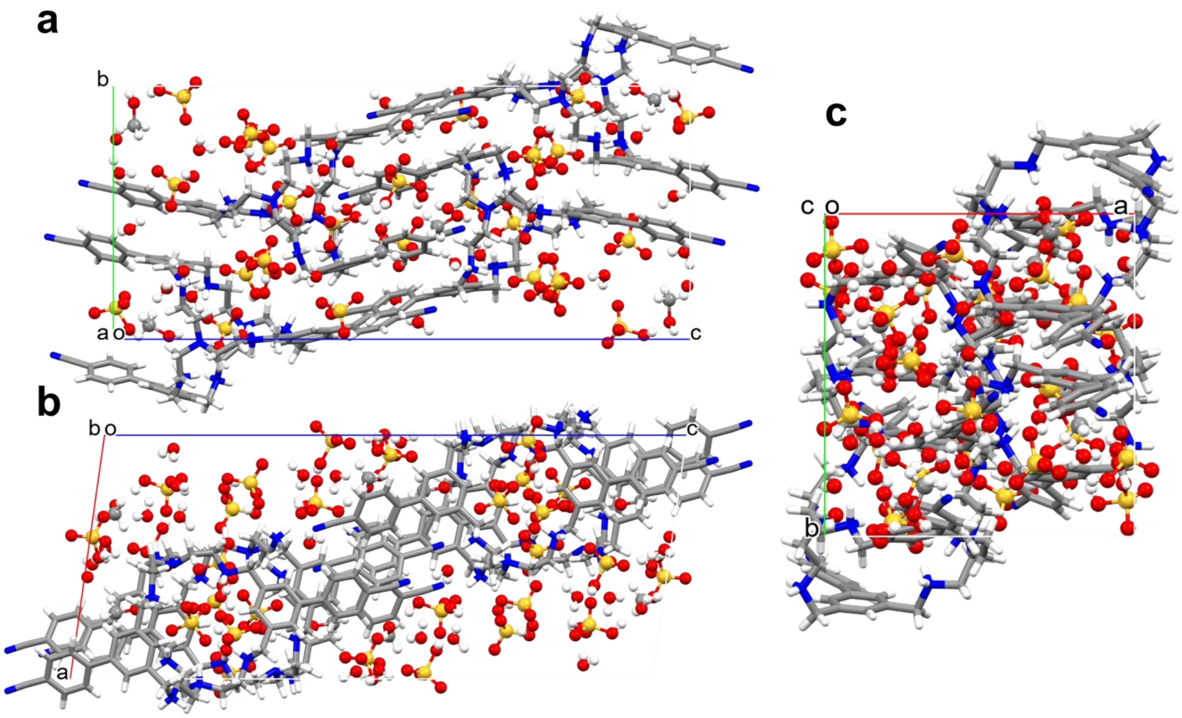

Figure S34. Crystal structure of Cage1-H_2SO_4 viewed along (a) *a*, (b) *b*, and (c) *c* axis. The protonated cages are shown in stick representation, while water and anions are shown in ball-and-stick representation. Element color code: yellow, S; red, O; blue, N; white, H; grey, C.

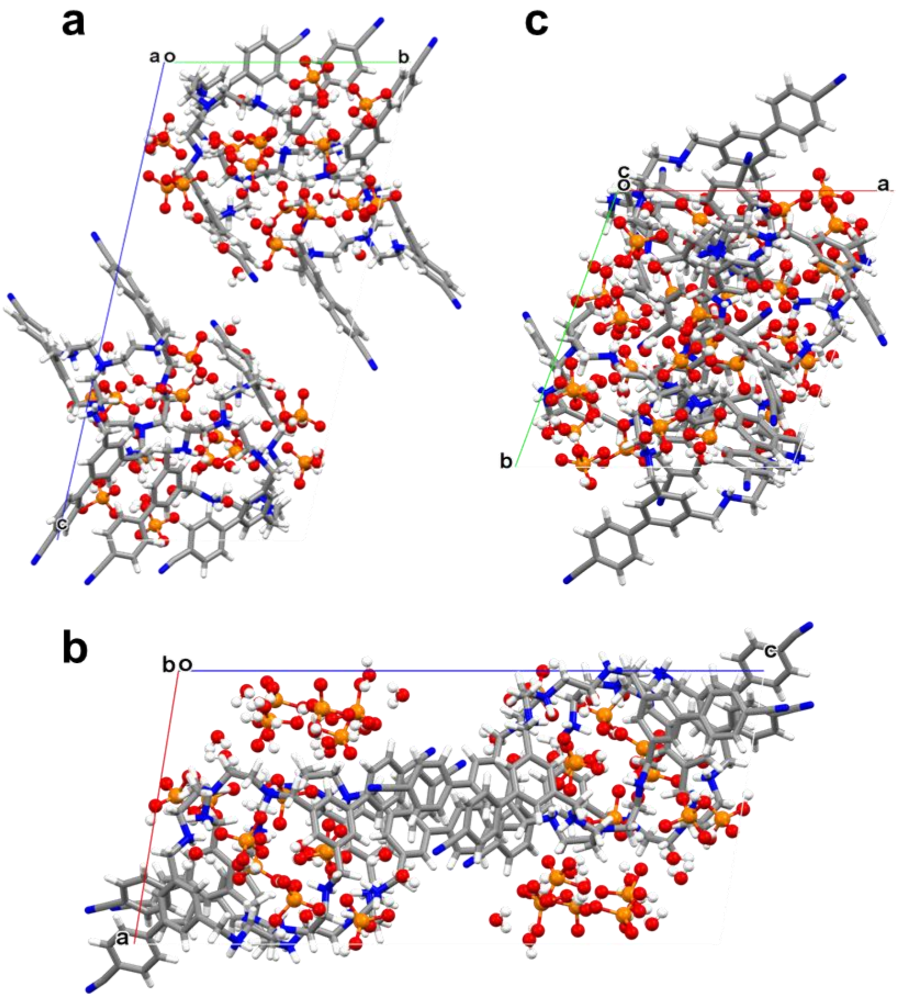

Figure S35. Crystal structure of Cage1-H_3PO_4 viewed along (a) *a*, (b) *b*, and (c) *c* axis. The protonated cages are shown in stick representation, while water and anions are shown in ball-and-stick representation. Element color code: orange, P; red, O; blue, N; white, H; grey, C.

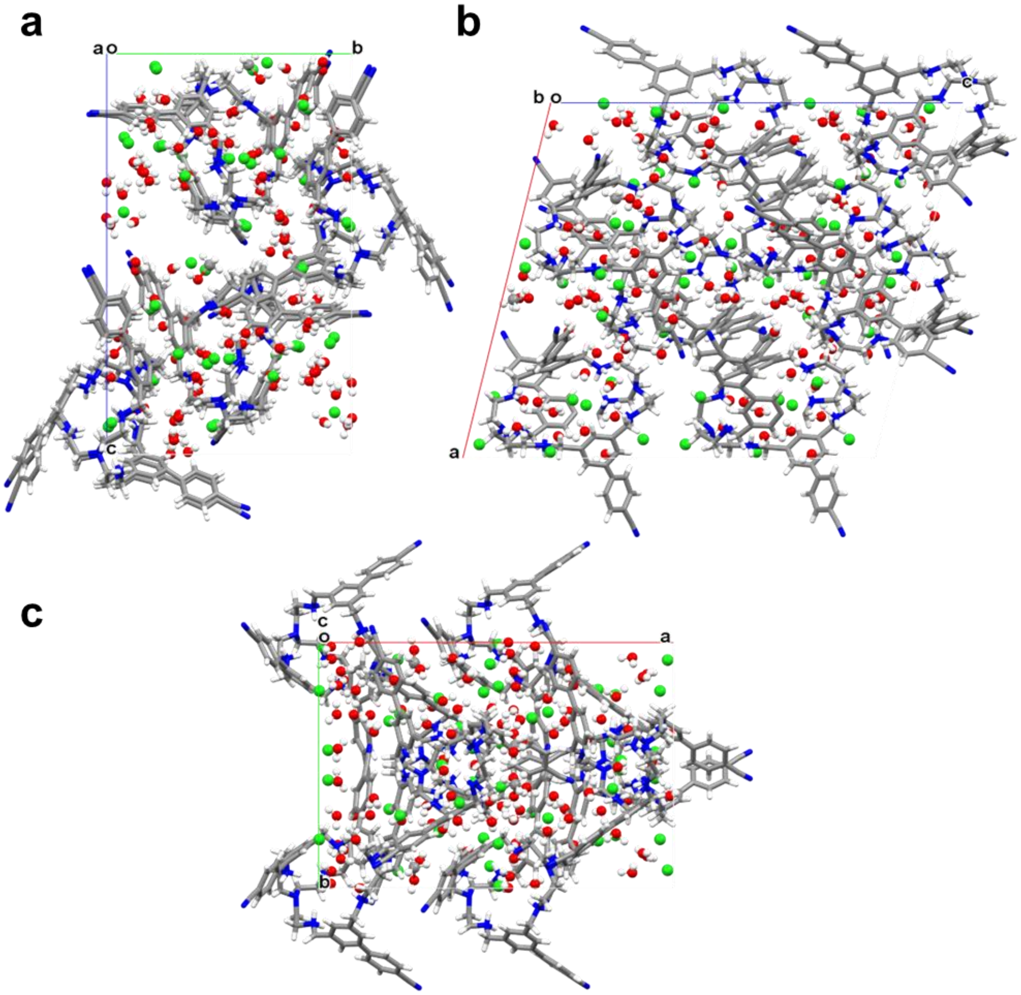

Figure S36. Crystal structure of Cage1-HCl viewed along (a) *a*, (b) *b*, and (c) *c* axis. The protonated cages are shown in stick representation, while water and anions are shown in ball-and-stick representation. Element color code: green, Cl; red, O; blue, N; white, H; grey, C.

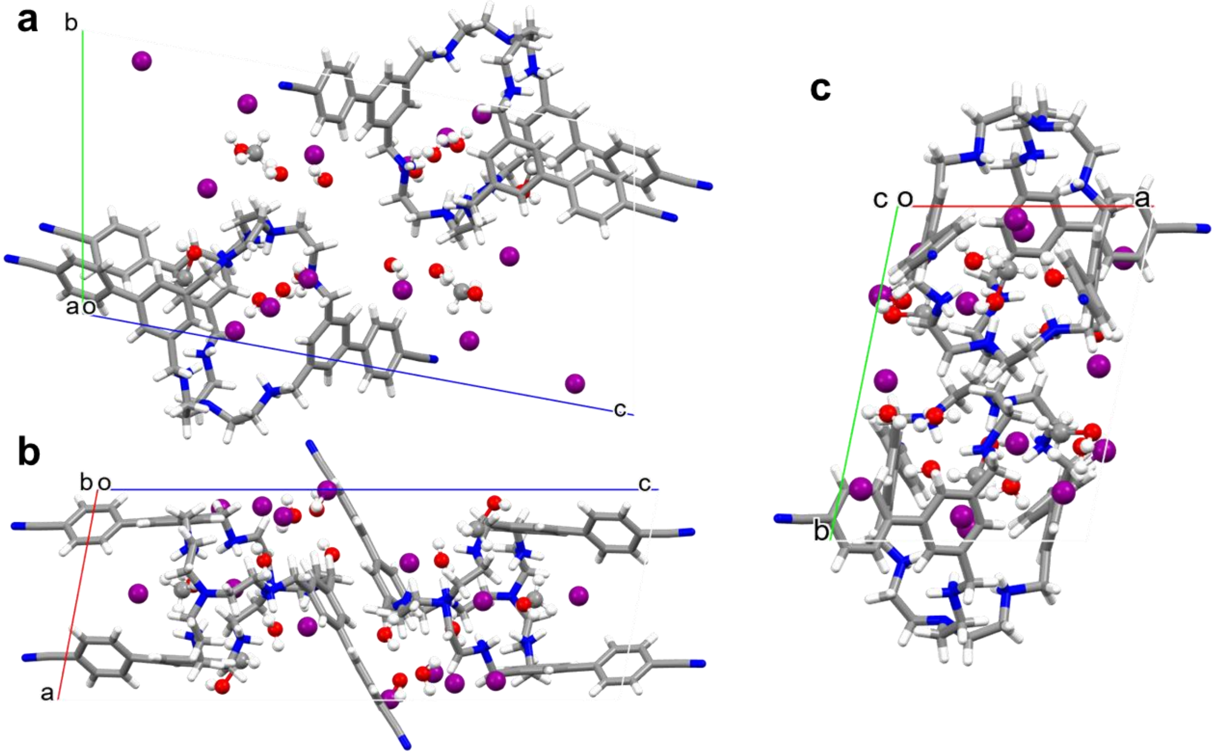

Figure S37. Crystal structure of Cage1-HI viewed along (a) *a*, (b) *b*, and (c) *c* axis. The protonated cages are shown in stick representation, while water and anions are shown in ball-and-stick representation. Element color code: purple, I; red, O; blue, N; white, H; grey, C.

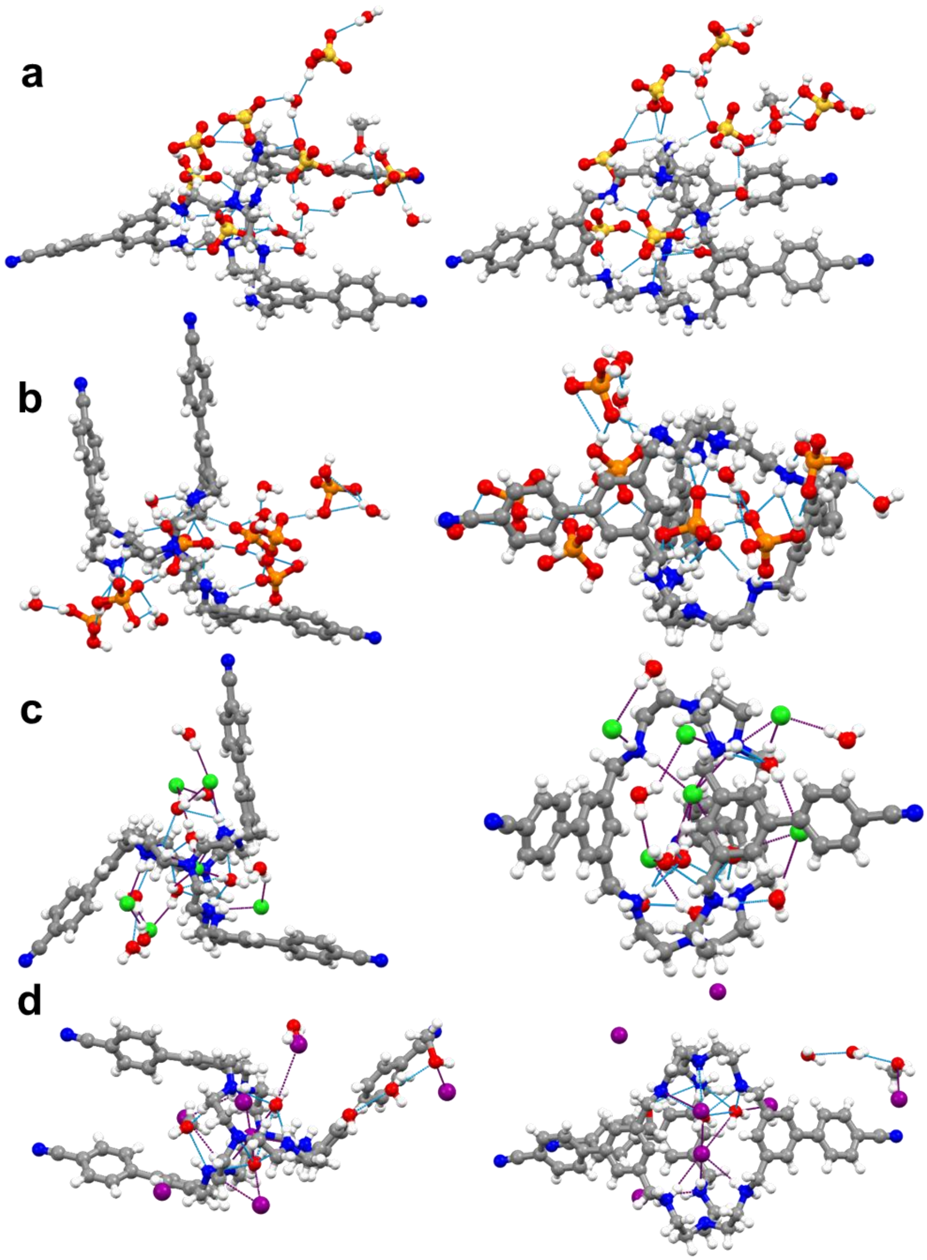

Figure S38. Hydrogen bond network around per protonated cage: top view and side view in (a) Cage1-H_2SO_4, (b) Cage1-H_3PO_4, (c) Cage1-HCl and (d) Cage1-HI. Element color code: red, O; yellow, S; orange, P; blue, N; white, H; grey, C; green, Cl; purple, I. Hydrogen bond color code (dashed line): cyan, O/N···H; purple, Cl/I···H.

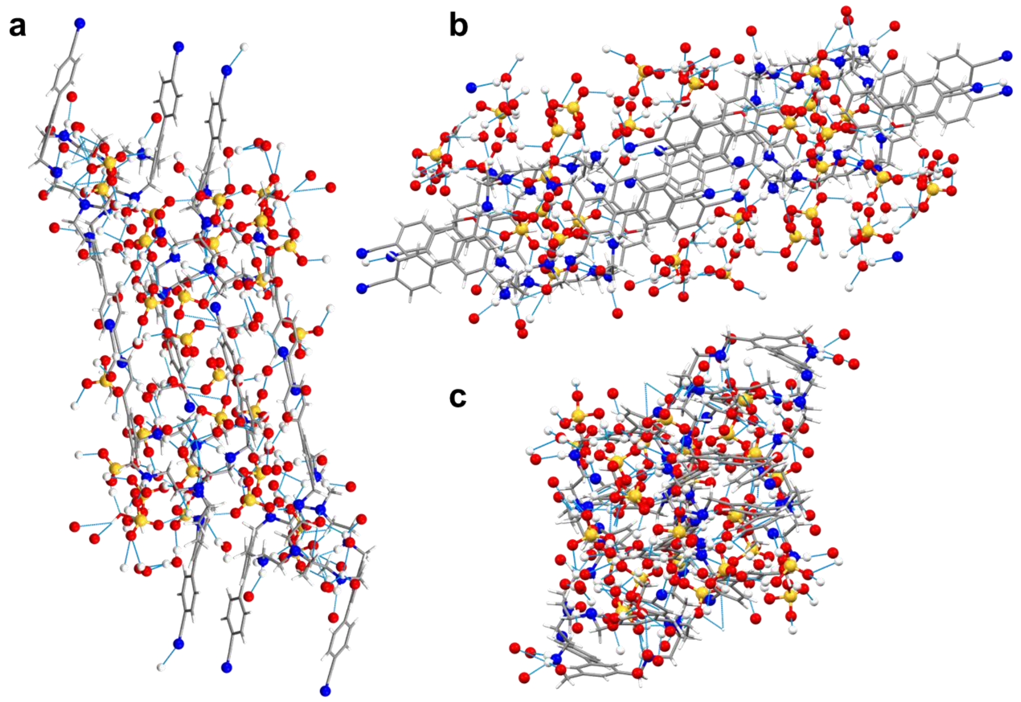

Figure S39. Hydrogen bond network in Cage1-H_2SO_4 cell viewed along (a) *a*, (b) *b*, and (c) *c* axis. The cage skeleton is shown in stick representation, while its -N/-NH_2 hydrogen bonding sites, water molecules, and the anions are shown in ball-and-stick representation. Element color code: red, O; yellow, S; blue, N; white, H; grey, C. Hydrogen bond (dashed line) is shown in cyan.

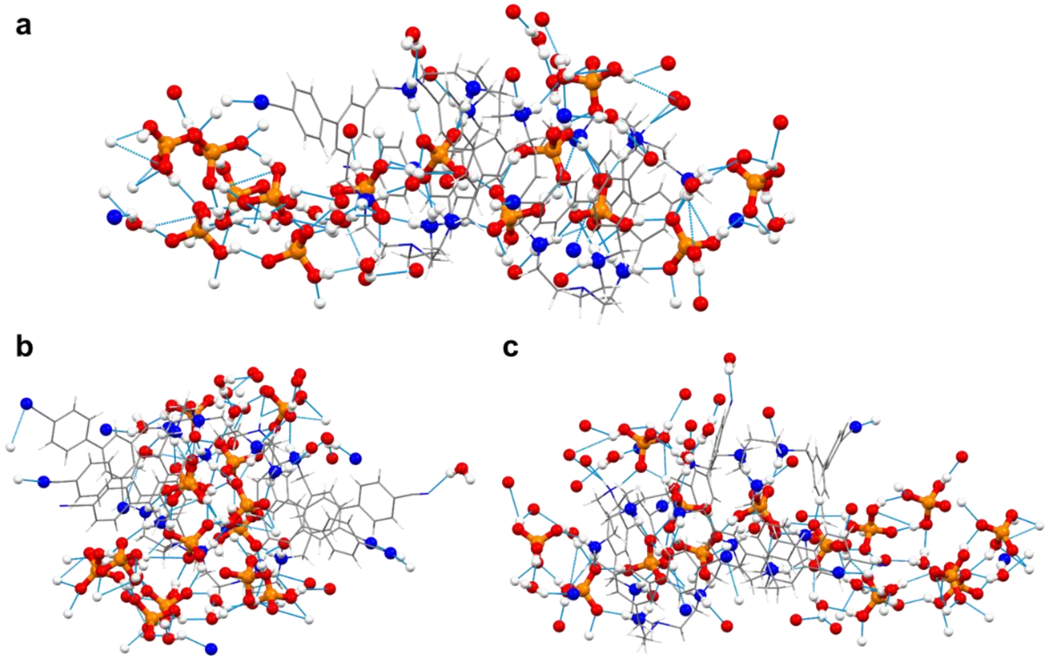

Figure S40. Hydrogen bond network in Cage1-H_3PO_4 cell viewed along (a) *a*, (b) *b*, and (c) *c* axis. The cage skeleton is shown in stick representation, while its -N/-NH_2 hydrogen bonding sites, water molecules, and the anions are shown in ball-and-stick representation. Element color code: red, O; orange, P; blue, N; white, H; grey, C. Hydrogen bond (dashed line) is shown in cyan.

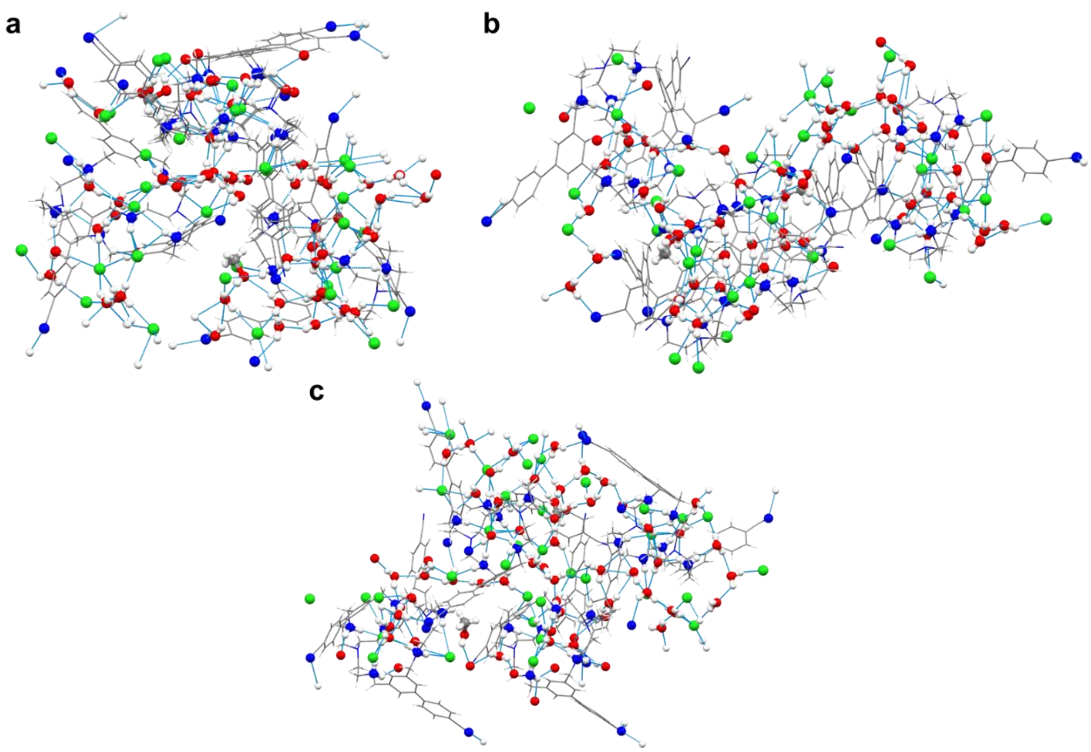

Figure S41. Hydrogen bond network in Cage1-HCl cell viewed along (a) *a*, (b) *b*, and (c) *c* axis. The cage skeleton is shown in stick representation, while its -N/-NH_2 hydrogen bonding sites, water molecules, and the anions are shown in ball-and-stick representation. Element color code: red, O; green, Cl; blue, N; white, H; grey, C. Hydrogen bond (dashed line) is shown in cyan.

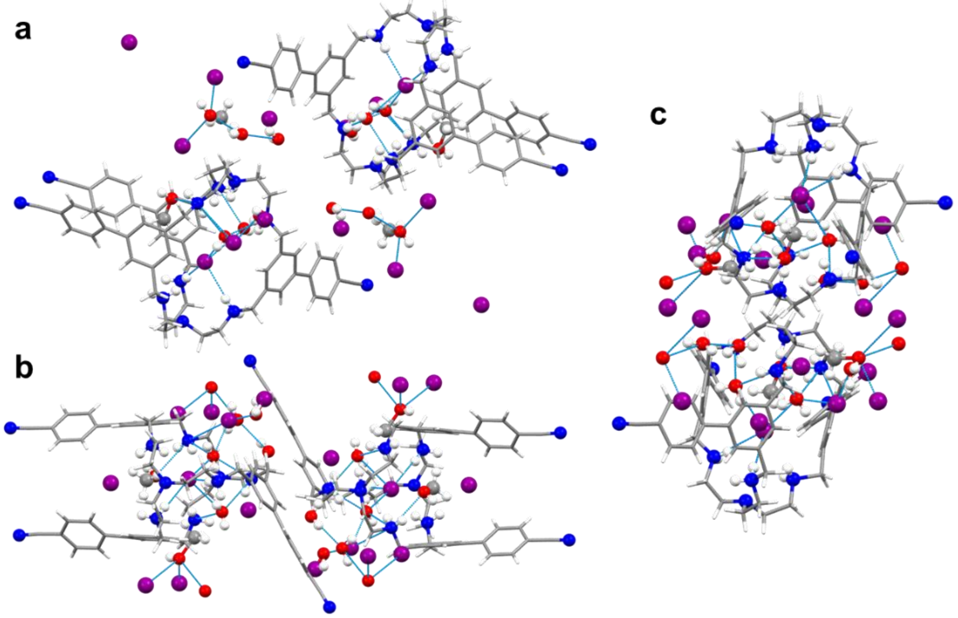

Figure S42. Hydrogen bond network in Cage1-HI cell viewed along (a) *a*, (b) *b*, and (c) *c* axis. The cage skeleton is shown in stick representation, while its -N/-NH_2 hydrogen bonding sites, water molecules, and the anions are shown in ball-and-stick representation. Element color code: red, O; purple, I; blue, N; white, H; grey, C. Hydrogen bond (dashed line) is shown in cyan.

Table S9. Hydrogen-bond distance details in Cage1-H_2SO_4 cell.

Number	Atom1	Atom2	Length
**1**	O2	O10	2.645
**2**	O1	N1	2.727
**3**	O1	N4	2.823
**4**	O4	N2	2.685
**5**	O4	N6	2.896
**6**	O3	N5	2.697
**7**	O2	N7	3.010
**8**	O3	O36	2.773
**9**	O9	O8	2.460
**10**	O12	N7	2.657
**11**	O11	N6	2.833
**12**	O10	N4	2.812
**13**	O7	N3	3.035
**14**	O5	N8	2.747
**15**	O6	N7	2.865
**16**	O21	N3	2.811
**17**	O24	O31	2.464
**18**	O22	O32	2.434
**19**	O21	O29	2.892
**20**	N4	O13	2.765
**21**	N3	O18	2.695
**22**	N6	O36	2.790
**23**	N5	O30	2.785
**24**	N8	O35	2.723
**25**	O16	O29	2.599
**26**	O14	O34	2.659
**27**	O15	O33	2.794
**28**	O27	O35	2.810
**29**	O28	O31	2.814
**30**	O17	O29	2.656
**31**	O30	O32	2.749
**32**	O35	O32	2.632
**33**	O34	O33	2.189
**34**	O34	O26A	2.176

Table S10. Hydrogen-bond distance details in Cage1-H_3PO_4 cell.

Number	Atom1	Atom2	Length
**1**	O1	O34	2.566
**2**	O32	O36	2.541
**3**	O32	O38	2.559
**4**	O31	N18	2.664
**5**	O1	O03U	2.824
**6**	O31	O05K	2.573
**7**	O3	O131	2.574
**8**	O35	O7	2.562
**9**	O33	O40	2.579
**10**	O35	N13	2.802
**11**	O34	N11	2.766
**12**	O35	O03L	2.96
**13**	O37	O17	2.495
**14**	O142	N22	2.699
**15**	O143	N21	2.621
**16**	O143	N23	2.688
**17**	O38	N18	2.806
**18**	O142	O01K	2.761
**19**	O4	O19	2.488
**20**	O6	O20	2.509
**21**	O5	N103	2.758
**22**	O10	O05P	2.352
**23**	O2	O13	2.529
**24**	O26	O013	3.003
**25**	O26	N10	2.565
**26**	O9	N25	2.901
**27**	O9	N11	2.938
**28**	O13	O16	2.606
**29**	O14	O17	2.607
**30**	O12	N9	2.935
**31**	O11	O124	2.518
**32**	O12	O42	2.665
**33**	O15	O21	2.558
**34**	O18	O22	2.575
**35**	O18	N26	2.709
**36**	O19	N2	2.773
**37**	O20	O05Z	2.885
**38**	O23	N13	2.609
**39**	O40	N23	2.749
**40**	O39	O04W	2.626
**41**	O24	O41	2.860
**42**	O013	N9	2.850
**43**	N39	O124	2.645

**44**	N8	O04W	2.960
**45**	N39	O135	2.643
**46**	N25	O136	2.815
**47**	N1	O05R	2.881
**48**	N20	O122	2.891
**49**	N27A	O01K	2.823
**50**	N26	O01K	2.736
**51**	N103	O01K	2.731
**52**	N20	O129	2.634
**53**	N22	O139	2.741
**54**	N24	O44	3.044
**55**	O122	O05K	2.933
**56**	O28	O134	2.715
**57**	O27	O42	2.842
**58**	O03L	O41	2.983
**59**	O03U	O05M	2.874
**60**	O03U	O132	2.666
**61**	O05K	O129	2.678
**62**	O05M	O30	2.933
**63**	O05M	O45	2.886
**64**	O05Z	O25	2.613
**65**	O05Z	O05P	2.528
**66**	O130	O139	2.634
**67**	O131	O05P	2.553
**68**	O133	O138	2.678
**69**	O136	O8	2.479
**70**	O137	O05R	2.429
**71**	O138	O29	2.650
**72**	O05R	O44	2.981
**73**	O42	O45	2.600

Table S11. Hydrogen-bond distance details in Cage1-HCl cell.

Number	Atom1	Atom2	Length
**1**	Cl01	O00S	3.188
**2**	Cl01	O00W	2.906
**3**	Cl01	N011	2.877
**4**	Cl01	N013	3.235
**5**	Cl02	N012	3.257
**6**	Cl02	N02E	3.032
**7**	Cl02	O01H	3.145
**8**	Cl03	N01P	3.221
**9**	Cl03	N02T	2.953
**10**	Cl03	O019	3.084
**11**	Cl04	O01F	3.124
**12**	Cl04	O01Z	3.068
**13**	Cl04	O08N	3.05
**14**	Cl05	N01O	2.855
**15**	Cl05	N02D	3.222
**16**	Cl05	O01L	3.236
**17**	Cl05	O04P	2.874
**18**	Cl06	N00Y	3.011
**19**	Cl06	O01X	3.121
**20**	Cl07	N01E	3.147
**21**	Cl07	O01Q	3.19
**22**	Cl08	O00O	3.026
**23**	Cl08	O01B	3.065
**24**	Cl08	O01Z	3.217
**25**	Cl09	N00R	3.078
**26**	Cl0A	O066	3.056
**27**	Cl0B	N02K	3.055
**28**	Cl0C	O030	2.813
**29**	Cl0D	O015	3.149
**30**	Cl0D	O033	2.961
**31**	Cl0E	N022	3.186
**32**	Cl0F	O00N	3.122
**33**	Cl0F	O014	3.019
**34**	Cl0F	O033	3.201
**35**	Cl0G	N05V	3.117
**36**	Cl0G	O010	2.735
**37**	Cl0H	O014	3.165
**38**	Cl0H	O4	2.979
**39**	Cl0I	N02T	3.209
**40**	Cl0I	O01I	3.079
**41**	Cl0I	O05W	2.939
**42**	Cl0I	O078	3.117
**43**	Cl0J	N01P	2.983

**44**	Cl0J	O05D	3.248
**45**	Cl0K	O00Q	2.961
**46**	Cl0K	O01B	3.174
**47**	Cl0K	O1	3.053
**48**	O00M	O3	2.752
**49**	O00M	O5	2.647
**50**	O00N	O01I	3.02
**51**	O00O	N01M	3.05
**52**	O00P	N00X	2.893
**53**	O00P	O01I	2.951
**54**	O00Q	N01C	2.748
**55**	O00Q	Cl3	3.265
**56**	O00Q	O09E	2.888
**57**	N00X	O015	2.597
**58**	N08M	O017	2.925
**59**	N012	O01T	2.694
**60**	N01Y	O01V	3.063
**61**	N02E	O03Y	2.835
**62**	N01Y	O04K	2.895
**63**	N02S	O06Q	3.053
**64**	O00S	N05V	2.635
**65**	O00S	O010	2.798
**66**	N01K	O01D	3.054
**67**	N024	O01D	2.602
**68**	N01C	O01F	2.569
**69**	N01K	O01J	2.848
**70**	N08S	O074	2.934
**71**	N02P	O4	2.879
**72**	O00V	N045	2.702
**73**	N013	O017	3.004
**74**	N011	O01G	2.871
**75**	N06E	O01V	2.809
**76**	O010	O01Q	2.939
**77**	O010	O02G	2.747
**78**	O014	N01A	3.052
**79**	O015	O01H	2.987
**80**	O016	O01J	2.528
**81**	O019	O01F	2.956
**82**	O019	Cl3	2.721
**83**	N08F	O01D	2.779
**84**	N08C	O021	2.675
**85**	N01O	O05A	2.895
**86**	N02D	O074	2.864
**87**	O01G	O01Z	2.817
**88**	O01H	O01V	3.015
**89**	O01H	Cl2	2.813

**90**	O01L	O5	2.76
**91**	O01T	O043	2.589
**92**	O01X	O08R	2.677
**93**	Cl2	O04F	3.207
**94**	O02G	Cl3	2.969
**95**	O030	O060	2.594
**96**	O033	O05A	2.816
**97**	O03H	O043	2.868
**98**	O03H	O09E	2.643
**99**	O03Y	O1	2.979
**100**	O043	O06Q	2.815
**101**	O04F	O5	2.71
**102**	O04K	O05C	2.556
**103**	O066	O06Q	2.602
**104**	Cl3	O09E	2.258
**105**	O078	O08N	2.495
**106**	O08N	O096	2.67
**107**	O08R	O3	2.378
**108**	O1	O2	2.895

Table S12. Hydrogen-bond distance details in Cage1-HI cell.

Number	Atom1	Atom2	Length
**1**	O7	O3	2.891
**2**	N7	O1	2.751
**3**	N7	I3	3.459
**4**	N6	O1	2.859
**5**	N3	I1	3.49
**6**	N4	I1	3.51
**7**	N6	O2	2.871
**8**	N8	O2	2.896
**9**	N7	O5	2.931
**10**	O1	I1	2.729
**11**	I1	O2	3.366
**12**	O3	O7	2.747
**13**	I1	O1	2.73
**14**	O7	I4	2.532

Calculations and Simulations

All the calculations and simulations were performed based on the crystal structures of Cage1-H_2SO_4, Cage1-H_3PO_4, Cage1-HCl and Cage1-HI. The density functional theory (DFT) calculations were performed at the B3LYP-D3(BJ)/6-31G(d) level by G16 package.[40] Independent gradient model based on Hirshfeld partition of molecular density (IGMH) analysis were used to visualize the supramolecular interactions in the host-guest systems. The Multiwfn software was used for the calculation and VMD packages were applied for visualization of IGMH and molecular electrostatic potential maps.[41] $Sign(\lambda_2)\rho$ colored isosurfaces of $\delta g^{inter} = 0.01$ a.u. of corresponding to IGMH analyses.

The molecular Hirshfeld maps with the fingerprint (2D) were calculated by using Crystal-Explorer software.[42] Hirshfeld surface analysis are assessed to obtain quantitative and qualitative insights into diverse type of intra or intermolecular interactions in a crystalline form of the researched compound. The Hirshfeld surface analysis is specified in the space occupied by a molecule in its crystal system based on the localized electron density distribution simulated around spherical atom. The Hirshfeld surface *via* fingerprint plot is a powerful method to calculate the intermolecular interactions and show it with well-defined color codes.

Molecular dynamics (MD) simulations were performed with the CP2K code based on crystal structures of Cage1-H_2SO_4, Cage1-H_3PO_4, Cage1-HCl and Cage1-HI, which uses a mixed Gaussian/plane-wave basis set.[43-44] All DFT calculations made use of the Perdew-Burke-Ernzerhof (PBE) exchange-correlation functional with semi-empirical dispersion corrections to the energies and gradients from the DFT-D3 method, the MOLOPT basis sets of the double z quality, together with the Goedecker–Teter–Hutter pseudopotentials.[45-47] AIMD simulations within the Born-Oppenheimer approximation were performed in the canonical (NVT) ensemble. A timestep of 1 fs was used for the integration of the equation of motion, and the simulations were run for 500 fs (500 AIMD steps following equilibration run that has a strong thermostat coupling). The temperature of the AIMD simulation was 500 K, which was controlled by canonical sampling through velocity rescaling thermostat using a time constant of 100 fs.

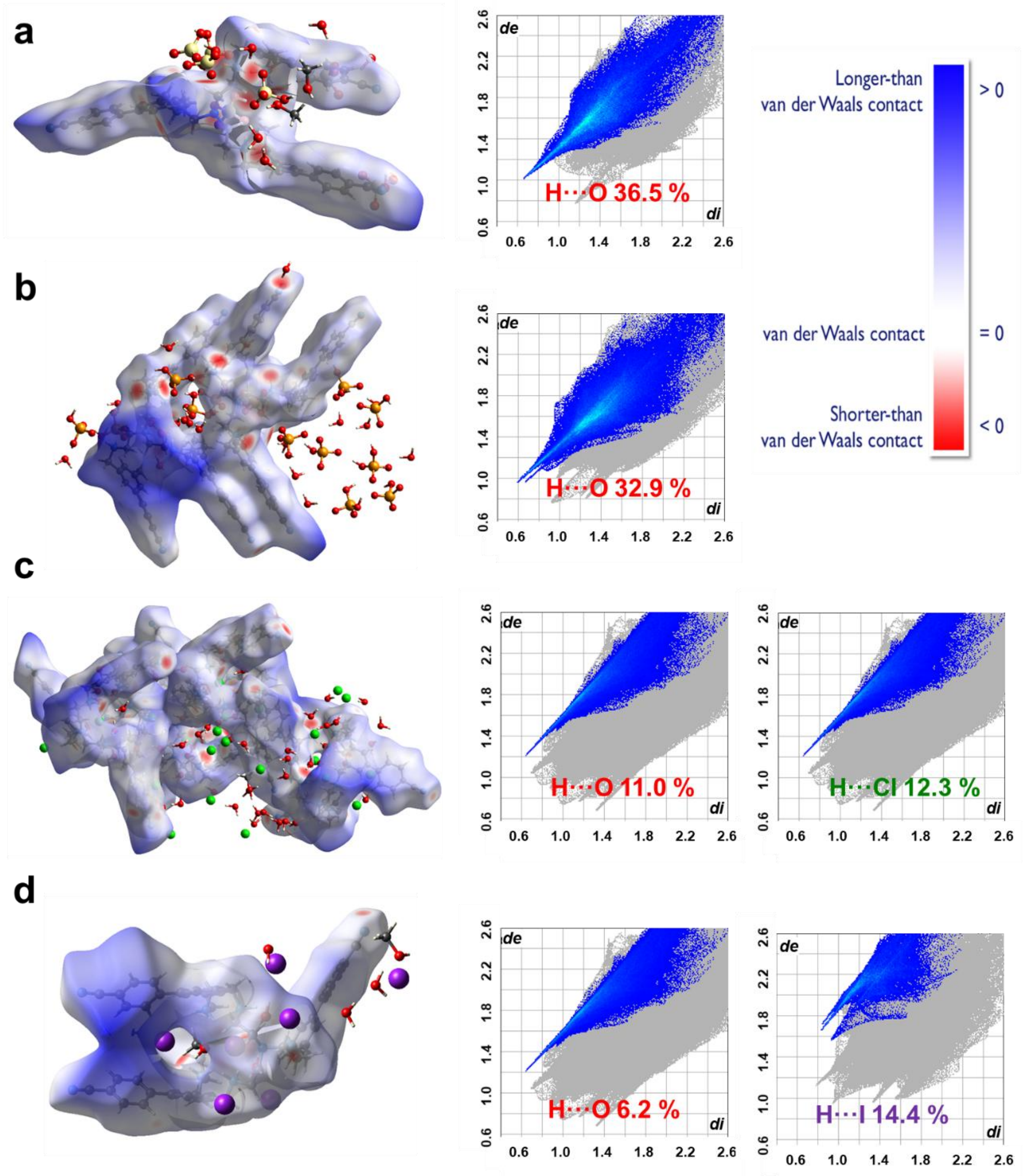

Figure S43. Hirshfeld surface analysis. Hirshfeld surface mapped with d_{norm} of (a) Cage1-H_2SO_4, (b) Cage1-H_3PO_4, (c) Cage1-HCl, and (d) Cage1-HI with their 2D fingerprint plots displaying the percentage contributions of H···O, H···Cl/I in crystals. X···Y: X belongs to the atom of the cage, Y belongs to atoms outside the cage.

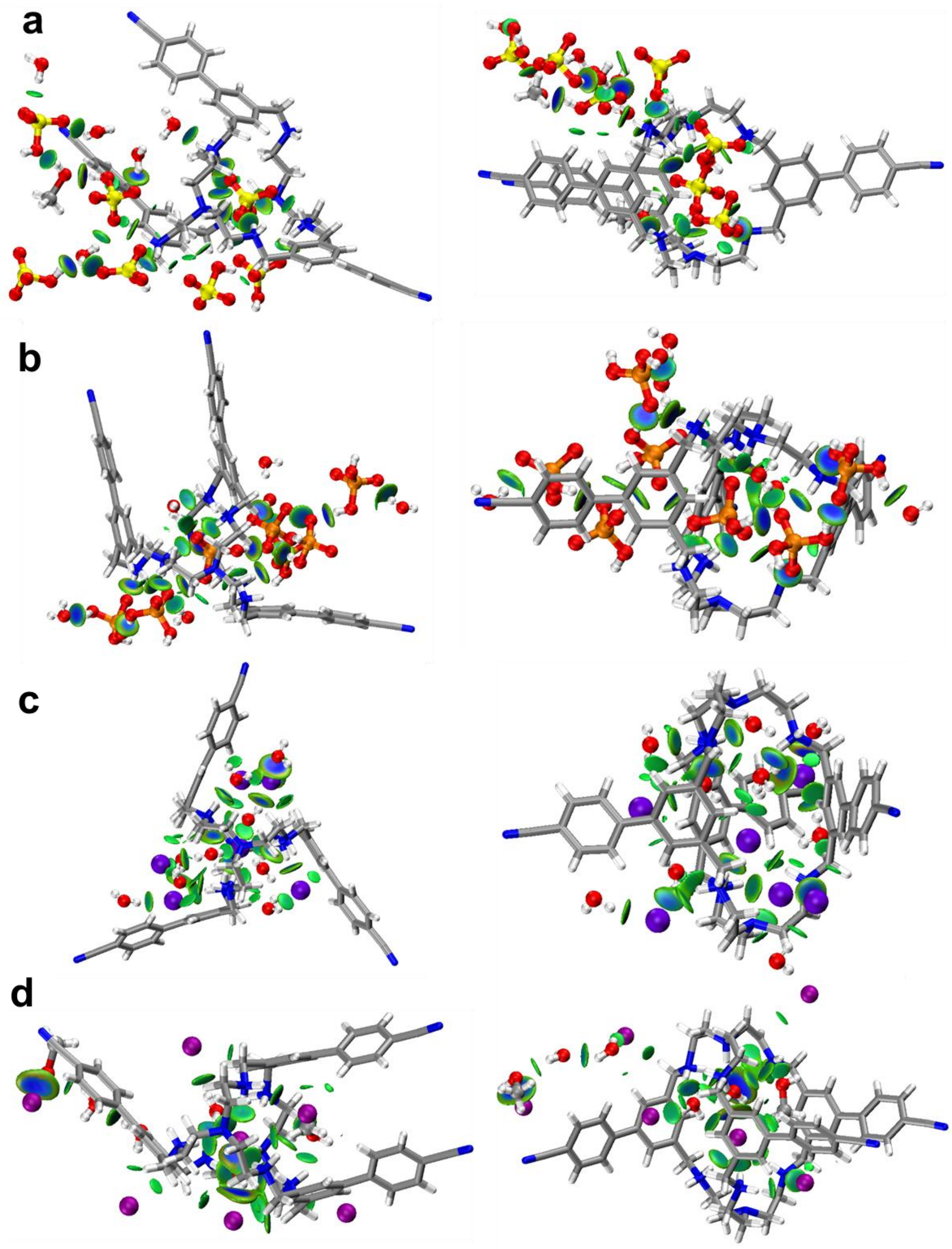

Figure S44. IGMH maps (top and side views) of (a) Cage1-H_2SO_4, (b) Cage1-H_3PO_4, (c) Cage1-HCl, and (d) Cage1-HI highlighting supramolecular interactions (green region), especially hydrogen bonding (blue region). *Sign(λ_2)ρ* colored isosurfaces of δg^{inter} = 0.01 a.u. of corresponding to IGMH analyses. The cage is shown in stick mode, other species are shown in ball-stick mode. Element color code: Red, O; blue, N; grey, C; white, H; yellow, S; orange, P; violet, Cl; purple, I.

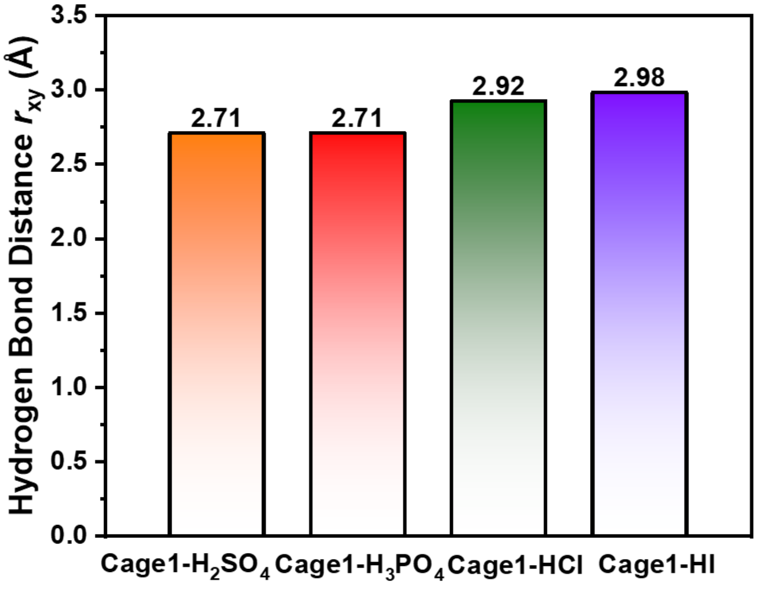

Figure S45. Averaged hydrogen bond distance (r_{XY} distance, X/Y means donor/acceptor atom, respectively) in Cage1-H_2SO_4, Cage1-H_3PO_4, Cage1-HCl and Cage1-HI crystals.

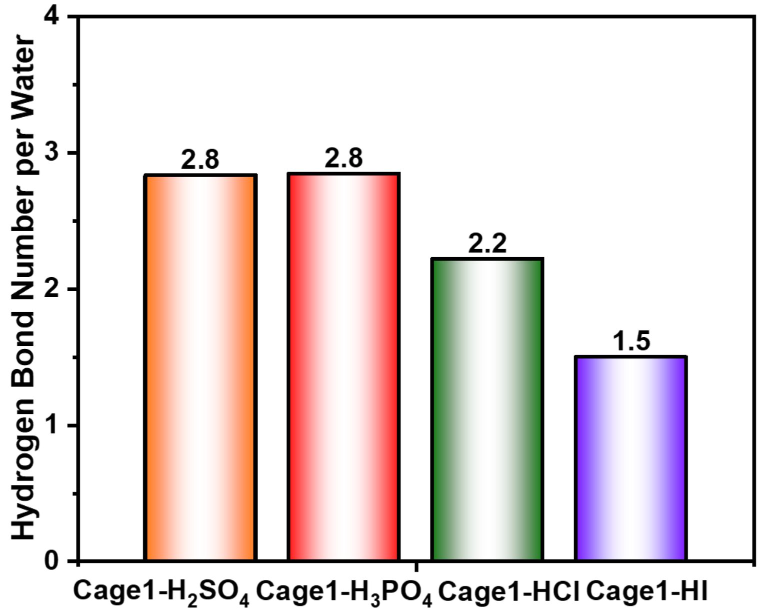

Figure S46. Averaged hydrogen bond number per water in Cage1-H_2SO_4, Cage1-H_3PO_4, Cage1-HCl and Cage1-HI crystals.

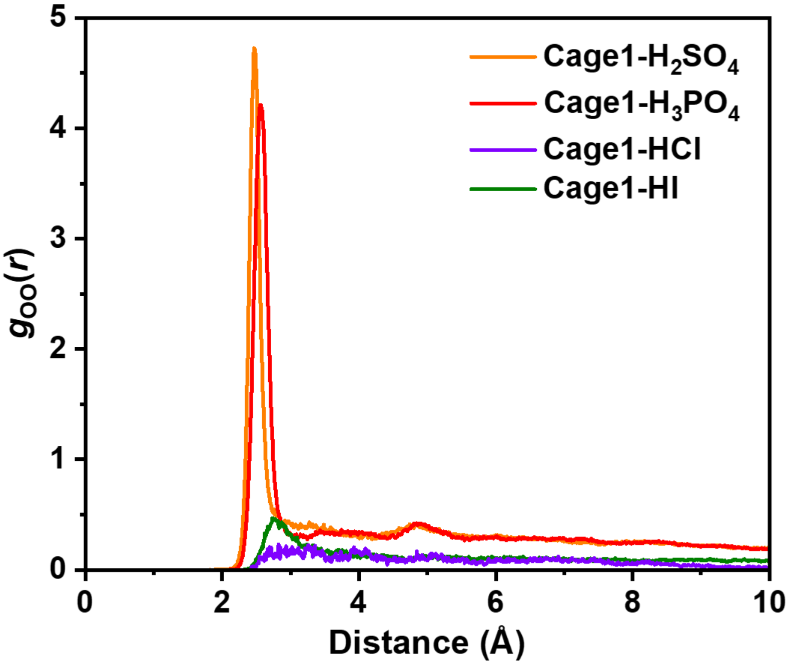

Figure S47. Calculated radial distribution functions $g_{oo}(r)$ representing oxygen-oxygen hydrogen bond distances in Cage1-H_2SO_4, Cage1-H_3PO_4, Cage1-HCl and Cage1-HI.

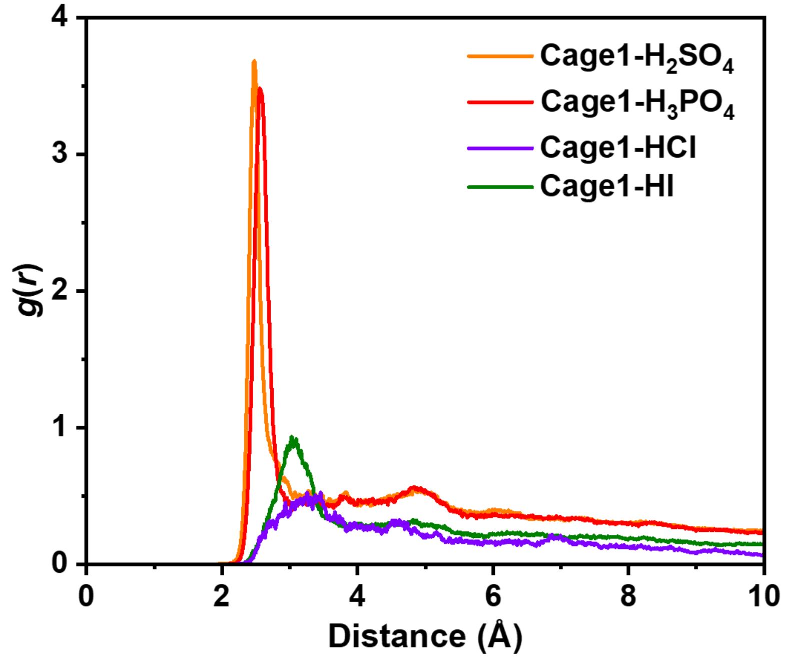

Figure S48. Calculated radial distribution functions $g(r)$ representing all hydrogen bond distances in Cage1-H_2SO_4, Cage1-H_3PO_4, Cage1-HCl and Cage1-HI.

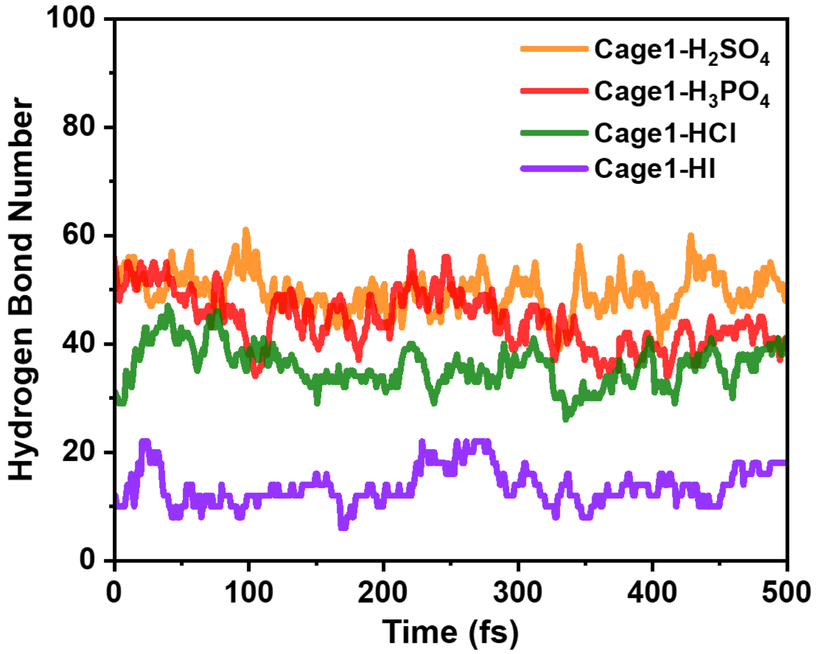

Figure S49. Calculated hydrogen bond numbers in Cage1-H_2SO_4, Cage1-H_3PO_4, Cage1-HCl and Cage1-HI.

References

(1) Blackburn, O. A.; Coe, B. J.; Helliwell, M.; Raftery, J. Syntheses, Structures, and Electronic and Optical Properties of Platinum(II) Complexes of 1,3-Bis(imino)benzene-Derived Pincer Ligands. *Organometallics* **2012**, *31* (15), 5307-5320.
(2) Yoon, M.; Suh, K.; Kim, H.; Kim, Y.; Selvapalam, N.; Kim, K. High and Highly Anisotropic Proton Conductivity in Organic Molecular Porous Materials. *Angew. Chem., Int. Ed.* **2011**, *50* (34), 7870-7873.
(3) Liu, M.; Chen, L.; Lewis, S.; Chong, S. Y.; Little, M. A.; Hasell, T.; Aldous, I. M.; Brown, C. M.; Smith, M. W.; Morrison, C. A. Three-dimensional protonic conductivity in porous organic cage solids. *Nat. Commun.* **2016**, *7* (1), 12750.
(4) Yang, Z.; Yu, C.; Ding, J.; Chen, L.; Liu, H.; Ye, Y.; Li, P.; Chen, J.; Wu, K. J.; Zhu, Q.-Y.; et al. A class of organic cages featuring twin cavities. *Nat. Commun.* **2021**, *12* (1), 6124.
(5) Yang, Z.; Zhang, N.; Lei, L.; Yu, C.; Ding, J.; Li, P.; Chen, J.; Li, M.; Ling, S.; Zhuang, X. Supramolecular Proton Conductors Self-Assembled by Organic Cages. *JACS Au* **2022**, *2* (4), 819-826.
(6) Wang, J.; Lin, W.; Chen, Z.; Nikolaeva, V. O.; Alimi, L. O.; Khashab, N. M. Smart touchless human–machine interaction based on crystalline porous cages. *Nat. Commun.* **2024**, *15* (1), 1575.
(7) Jiménez-García, L.; Kaltbeitzel, A.; Pisula, W.; Gutmann, J. S.; Klapper, M.; Müllen, K. Phosphonated Hexaphenylbenzene: A Crystalline Proton Conductor. *Angew. Chem., Int. Ed.* **2009**, *48* (52), 9951-9953.
(8) Phang, W. J.; Jo, H.; Lee, W. R.; Song, J. H.; Yoo, K.; Kim, B.; Hong, C. S. Superprotonic conductivity of a UiO - 66 framework functionalized with sulfonic acid groups by facile postsynthetic oxidation. *Angew. Chem., Int. Ed.* **2015**, *54* (17), 5142-5146.
(9) Kang, D. W.; Lim, K. S.; Lee, K. J.; Lee, J. H.; Lee, W. R.; Song, J. H.; Yeom, K. H.; Kim, J. Y.; Hong, C. S. Cost-Effective, High-Performance Porous-Organic-Polymer Conductors Functionalized with Sulfonic Acid Groups by Direct Postsynthetic Substitution. *Angew. Chem., Int. Ed.* **2016**, *55* (52), 16123-16126.
(10) Karmakar, A.; Illathvalappil, R.; Anothumakkool, B.; Sen, A.; Samanta, P.; Desai, A. V.; Kurungot, S.; Ghosh, S. K. Hydrogen - bonded organic frameworks (HOFs): a new class of porous crystalline proton - conducting materials. *Angew. Chem., Int. Ed.* **2016**, *55* (36), 10667-10671.
(11) Wei, Y.-S.; Hu, X.-P.; Han, Z.; Dong, X.-Y.; Zang, S.-Q.; Mak, T. C. W. Unique Proton Dynamics in an Efficient MOF-Based Proton Conductor. *J. Am. Chem. Soc.* **2017**, *139* (9), 3505-3512.
(12) Sasmal, H. S.; Aiyappa, H. B.; Bhange, S. N.; Karak, S.; Halder, A.; Kurungot, S.; Banerjee, R. Superprotonic Conductivity in Flexible Porous Covalent Organic Framework Membranes. *Angew. Chem., Int. Ed.* **2018**, *57* (34), 10894-10898.
(13) Xing, G.; Yan, T.; Das, S.; Ben, T.; Qiu, S. Synthesis of Crystalline Porous Organic Salts with High Proton Conductivity. *Angew. Chem., Int. Ed.* **2018**, *57* (19), 5345-5349.
(14) Wang, S.; Wahiduzzaman, M.; Davis, L.; Tissot, A.; Shepard, W.; Marrot, J.; Martineau-Corcos, C.; Hamdane, D.; Maurin, G.; Devautour-Vinot, S. A robust zirconium amino acid metal-organic framework for proton conduction. *Nat. Commun.* **2018**, *9* (1), 4937.
(15) Ranjeesh, K. C.; Illathvalappil, R.; Veer, S. D.; Peter, J.; Wakchaure, V. C.; Goudappagouda; Raj, K. V.; Kurungot, S.; Babu, S. S. Imidazole-Linked Crystalline Two-

Dimensional Polymer with Ultrahigh Proton-Conductivity. *J. Am. Chem. Soc.* **2019**, *141* (38), 14950-14954.
(16) Liang, H.-Q.; Guo, Y.; Shi, Y.; Peng, X.; Liang, B.; Chen, B. A Light-Responsive Metal–Organic Framework Hybrid Membrane with High On/Off Photoswitchable Proton Conductivity. *Angew. Chem., Int. Ed.* **2020**, *59* (20), 7732-7737.
(17) Qian, X.; Chen, L.; Yin, L.; Liu, Z.; Pei, S.; Li, F.; Hou, G.; Chen, S.; Song, L.; Thebo, K. H. CdPS3 nanosheets-based membrane with high proton conductivity enabled by Cd vacancies. *Science* **2020**, *370* (6516), 596-600.
(18) Tao, S.; Zhai, L.; Dinga Wonanke, A.; Addicoat, M. A.; Jiang, Q.; Jiang, D. Confining H3PO4 network in covalent organic frameworks enables proton super flow. *Nat. Commun.* **2020**, *11* (1), 1981.
(19) Sarango-Ramírez, M. K.; Lim, D.-W.; Kolokolov, D. I.; Khudozhitkov, A. E.; Stepanov, A. G.; Kitagawa, H. Superprotonic conductivity in metal–organic framework via solvent-free coordinative urea insertion. *J. Am. Chem. Soc.* **2020**, *142* (15), 6861-6865.
(20) Im, Y.-K.; Lee, D.-G.; Noh, H.-J.; Yu, S.-Y.; Mahmood, J.; Lee, S.-Y.; Baek, J.-B. Crystalline Porphyrazine-Linked Fused Aromatic Networks with High Proton Conductivity. *Angew. Chem., Int. Ed.* **2022**, *61* (28), e202203250.
(21) Lu, Z.; Yang, C.; He, L.; Hong, J.; Huang, C.; Wu, T.; Wang, X.; Wu, Z.; Liu, X.; Miao, Z.; et al. Asymmetric Hydrophosphonylation of Imines to Construct Highly Stable Covalent Organic Frameworks with Efficient Intrinsic Proton Conductivity. *J. Am. Chem. Soc.* **2022**, *144* (22), 9624-9633.
(22) Otsubo, K.; Nagayama, S.; Kawaguchi, S.; Sugimoto, K.; Kitagawa, H. A Preinstalled Protic Cation as a Switch for Superprotonic Conduction in a Metal–Organic Framework. *JACS Au* **2022**, *2* (1), 109-115.
(23) He, Y.; Dong, J.; Liu, Z.; Li, M.-Q.; Hu, J.; Zhou, Y.; Xu, Z.; He, J. Dense Dithiolene Units on Metal–Organic Frameworks for Mercury Removal and Superprotonic Conduction. *ACS Appl. Mater. Interfaces* **2022**, *14* (1), 1070-1076.
(24) Bai, X.-T.; Cao, L.-H.; Ji, C.; Zhao, F.; Chen, X.-Y.; Cao, X.-J.; Huang, M.-F. Ultra-High Proton Conductivity iHOF Based on Guanidinium Arylphosphonate for Proton Exchange Membrane Fuel Cells. *Chem. Mater.* **2023**, *35* (8), 3172-3180.
(25) Chen, S.; Ju, Y.; Zhang, H.; Zou, Y.; Lin, S.; Li, Y.; Wang, S.; Ma, E.; Deng, W.; Xiang, S. Photo Responsive Electron and Proton Conductivity within a Hydrogen - Bonded Organic Framework. *Angew. Chem., Int. Ed.* **2023**, *62* (34), e202308418.
(26) Choi, J. Y.; Stodolka, M.; Kim, N.; Pham, H. T.; Check, B.; Park, J. 2D conjugated metal-organic framework as a proton-electron dual conductor. *Chem* **2023**, *9* (1), 143-153.
(27) Guan, L.; Guo, Z.; Zhou, Q.; Zhang, J.; Cheng, C.; Wang, S.; Zhu, X.; Dai, S.; Jin, S. A highly proton conductive perfluorinated covalent triazine framework via low-temperature synthesis. *Nat. Commun.* **2023**, *14* (1), 8114.
(28) Hao, L.; Jia, S.; Qiao, X.; Lin, E.; Yang, Y.; Chen, Y.; Cheng, P.; Zhang, Z. Pore Geometry and Surface Engineering of Covalent Organic Frameworks for Anhydrous Proton Conduction. *Angew. Chem., Int. Ed.* **2023**, *62* (6), e202217240.
(29) Sharma, A.; Lim, J.; Lee, S.; Han, S.; Seong, J.; Bin Baek, S.; Soo Lah, M. Superprotonic Conductivity of MOFs Confining Zwitterionic Sulfamic Acid as Proton Source and Conducting Medium. *Angew. Chem., Int. Ed.* **2023**, *62* (29), e202302376.

(30) Sarango‐Ramírez, M. K.; Donoshita, M.; Yoshida, Y.; Lim, D. W.; Kitagawa, H. Cooperative Proton and Li‐ion Conduction in a 2D‐Layered MOF via Mechanical Insertion of Lithium Halides. *Angew. Chem., Int. Ed.* **2023**, *62* (19), e202301284.
(31) Li, X.-X.; Li, C.-H.; Hou, M.-J.; Zhu, B.; Chen, W.-C.; Sun, C.-Y.; Yuan, Y.; Guan, W.; Qin, C.; Shao, K.-Z.; et al. Ce-mediated molecular tailoring on gigantic polyoxometalate {Mo132} into half-closed {Ce11Mo96} for high proton conduction. *Nat. Commun.* **2023**, *14* (1), 5025.
(32) Lupa-Myszkowska, M.; Oszajca, M.; Matoga, D. From non-conductive MOF to proton-conducting metal-HOFs: a new class of reversible transformations induced by solvent-free mechanochemistry. *Chem. Sci.* **2023**, *14* (48), 14176-14181.
(33) Zou, W.; Jiang, G.; Zhang, W.; Zhang, L.; Cui, Z.; Song, H.; Liang, Z.; Du, L. Hierarchically macro–microporous covalent organic frameworks for efficient proton conduction. *Adv. Funct. Mater.* **2023**, *33* (18), 2213642.
(34) Ami, T.; Oka, K.; Kitajima, S.; Tohnai, N. Highly Fluorinated Nanospace in Porous Organic Salts with High Water Stability/Capability and Proton Conductivity. *Angew. Chem., Int. Ed.* **2024**, *63* (37), e202407484.
(35) O'Shaughnessy, M.; Lim, J.; Glover, J.; Neale, A. R.; Day, G. M.; Hardwick, L. J.; Cooper, A. I. Nonmetal Organic Frameworks Exhibit High Proton Conductivity. *J. Am. Chem. Soc.* **2025**, *147* (18), 15429-15434.
(36) Sheldrick, G. *SADABS*; University of Gottingen, Germany, 2008.
(37) Sheldrick, G. M. Crystal structure refinement with SHELXL. *Acta Crystallogr., Sect. C: Struct. Chem.* **2015**, *71* (1), 3-8.
(38) Dolomanov, O. V.; Bourhis, L. J.; Gildea, R. J.; Howard, J. A.; Puschmann, H. OLEX2: a complete structure solution, refinement and analysis program. *J. Appl. Crystallogr.* **2009**, *42* (2), 339-341.
(39) Barbour, L. J. X-Seed—A software tool for supramolecular crystallography. *J. Supramol. Chem.* **2001**, *1*, 189-191.
(40) *Gaussian 16 Rev. C.01*; Wallingford, CT, 2016. (accessed.
(41) Lu, T.; Chen, F. Multiwfn: a multifunctional wavefunction analyzer. *J. Comput. Chem.* **2012**, *33* (5), 580-592.
(42) Spackman, P. R.; Turner, M. J.; McKinnon, J. J.; Wolff, S. K.; Grimwood, D. J.; Jayatilaka, D.; Spackman, M. A. CrystalExplorer: a program for Hirshfeld surface analysis, visualization and quantitative analysis of molecular crystals. *J. Appl. Crystallogr.* **2021**, *54* (3), 1006-1011.
(43) Hutter, J.; Iannuzzi, M.; Schiffmann, F.; VandeVondele, J. cp2k: atomistic simulations of condensed matter systems. *Wiley Interdisciplinary Reviews: Computational Molecular Science* **2014**, *4* (1), 15-25.
(44) Kühne, T. D.; Iannuzzi, M.; Del Ben, M.; Rybkin, V. V.; Seewald, P.; Stein, F.; Laino, T.; Khaliullin, R. Z.; Schütt, O.; Schiffmann, F. CP2K: An electronic structure and molecular dynamics software package-Quickstep: Efficient and accurate electronic structure calculations. *J. Chem. Phys.* **2020**, *152* (19).
(45) Perdew, J. P.; Burke, K.; Ernzerhof, M. Generalized gradient approximation made simple. *Physical review letters* **1996**, *77* (18), 3865.
(46) Grimme, S.; Antony, J.; Ehrlich, S.; Krieg, H. A consistent and accurate ab initio parametrization of density functional dispersion correction (DFT-D) for the 94 elements H-Pu. *J. Chem. Phys.* **2010**, *132* (15).

(47) VandeVondele, J.; Hutter, J. Gaussian basis sets for accurate calculations on molecular systems in gas and condensed phases. *J. Chem. Phys.* **2007**, *127* (11).
